# Synergistic Efficacy of Gedatolisib and Darolutamide in Prostate Cancer to Overcome Resistance to Androgen-Targeted Therapy

**DOI:** 10.3390/ijms262411810

**Published:** 2025-12-06

**Authors:** Salmaan Khan, Jhomary Molden, Charles Iversrud, Donna Mattonen, Stefano Rossetti, Lance Laing

**Affiliations:** Celcuity, Inc., 16305 36th Ave N, Suite 100, Minneapolis, MN 55446, USA; skhan@celcuity.com (S.K.); jmolden@celcuity.com (J.M.); civersrud@celcuity.com (C.I.); dmattonen@celcuity.com (D.M.)

**Keywords:** prostate cancer, PI3K/AKT/mTOR pathway, gedatolisib, darolutamide

## Abstract

The oncogenic activation of the PI3K/AKT/mTOR (PAM) pathway, which is often associated with loss of PTEN, is an important adaptive mechanism to androgen-targeted therapy in castration-resistant prostate cancer (CRPC). The concomitant targeting of the PAM pathway and the androgen receptor (AR) pathway is a promising therapeutic strategy for CRPC. Many PAM pathway inhibitors only target one component of the PAM pathway, which can limit efficacy due to the activation of the uninhibited components. We previously showed that the multi-target pan-PI3K-mTORC1/2 inhibitor, gedatolisib, exerts greater growth-inhibitory effects than single-target PAM pathway inhibitors in prostate cancer (PC) cells, regardless of PTEN or AR status. In the present study, we investigated the molecular and cellular effects of gedatolisib in combination with darolutamide in both PTEN+ and PTEN-deficient PC cell lines, including AR+ PC cell lines adapted to long-term treatment with darolutamide. We found that the gedatolisib + darolutamide combination exerted greater anti-proliferative and cytotoxic effects than the single agents in most AR+ PC cell models, regardless of their PTEN status. The gedatolisib + darolutamide combination inhibited AR and PAM pathway activities, blocked cell cycle progression, induced apoptotic cell death, and reduced glucose and lipid metabolism. The drug combination was effective in both darolutamide-naïve and darolutamide-adapted cell lines, suggesting potential benefit in prostate tumors that progressed after androgen-targeted therapy. These results provide a strong rationale for clinical studies evaluating gedatolisib in combination with AR inhibitors in CRPC.

## 1. Introduction

Due to the significant role established for androgen signaling in prostate cancer (PC) progression, androgen deprivation therapy (ADT) has become a mainstay of advanced PC treatment. Surgical or chemical castration to suppress androgen synthesis in the testes is often combined with, or followed by, a more complete androgen blockade through inhibitors of androgen synthesis in other tissues or inhibitors of the androgen receptor (AR) function (e.g., enzalutamide, darolutamide). While these androgen-targeted therapies are initially effective, PC cells often develop resistance mechanisms that lead to castration-resistant prostate cancer (CRPC), which is often metastatic at diagnosis. Patients with metastatic CRPC (mCRPC) have a generally poor prognosis and relatively few therapeutic options [1].

Most CRPC tumors develop mechanisms that restore the AR signaling (e.g., AR overexpression, increase in AR splice variants, intra-tumoral androgen production via CYP17A1 or AKR1C3) to drive tumor progression despite treatment with androgen-targeted therapies [2]. While these mechanisms are common, PC cells also increasingly rely on alternative signaling pathways to promote cell survival and proliferation. The PI3K-AKT-mTOR (PAM) pathway is one of the most relevant pathways aberrantly activated during PC progression and androgen-targeted therapy resistance [3,4]. Dysregulation of the PAM pathway during PC progression has been associated with genetic alterations of PAM pathway genes, such as *PTEN*, which encodes a natural negative regulator of the PAM pathway, and *PIK3CA*, which encodes for the p110α catalytic subunit of PI3K [5]. Inactivation of the *PTEN* gene by deletion or mutation and loss of PTEN protein expression are especially frequent, with their prevalence increasing from an estimated 15–20% in primary PC up to 40–60% in CRPC and metastatic PC [6,7].

The increased activation of the PAM pathway in cancer cells couples tumor-promoting functions, such as cell cycle progression and cell survival, with metabolic adaptations (e.g., increased glycolysis and lipid synthesis) providing energy and biomolecules necessary for anabolic processes [8,9]. Given the reliance of cancer cells on the PAM pathway, this pathway is considered a promising therapeutic target in multiple cancer types [10]. Several inhibitors targeting single PAM pathway components (PI3Kα, AKT, mTORC1) have been approved by the FDA for treatment of hormone-driven breast cancer in combination with hormone therapy, but no PAM pathway inhibitors are currently indicated for hormone-driven prostate cancer.

**Figure 1:**
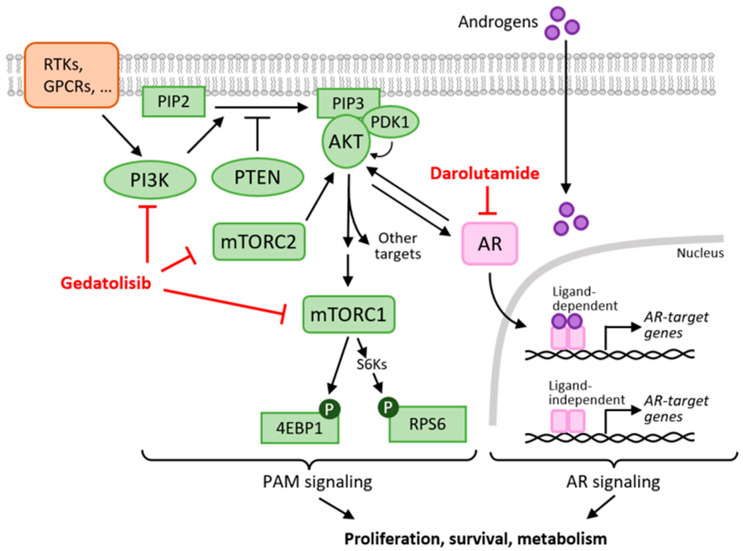
Crosstalk between the PI3K/AKT/mTOR (PAM) pathway and the Androgen Receptor (AR) pathway. The PAM pathway controls key cellular functions involved in PC initiation and progression, including cell metabolism, cell survival, and cell proliferation. PI3K, AKT, mTOR complex 1 (mTORC1), and mTOR complex 2 (mTORC2) are key components of the PAM pathway. The catalytic subunit of class I PI3Ks, p110, also has multiple isoforms (α, β, γ, δ). Upon stimulation by extracellular signals transduced by various membrane receptors (e.g., RTKs, GPCRs), PI3K converts phosphatidylinositol (4,5)-bisphosphate (PIP2) into phosphatidylinositol (3,4,5)-trisphosphate (PIP3). PIP3 accumulation leads to the activation of downstream targets, such as AKT, which controls multiple effectors (e.g., mTORC1). mTORC2, a downstream effector of PI3K, can further activate AKT, while the PTEN phosphatase can terminate PAM activation by converting PIP3 to PIP2. The AR pathway, through androgen-dependent and androgen-independent transcriptional regulation, also controls key cellular functions driving PC progression. The PAM and AR pathways are linked through multiple mechanisms that can lead to reciprocal regulation. Concomitant targeting of the PAM pathway (e.g., by gedatolisib) and the AR pathway (e.g., by darolutamide), shown in red, is a promising strategy for PC treatment. RTK = receptor tyrosine kinase; GPCR = G-protein-coupled receptors. Scheme based on [3,4].

The PAM pathway is interconnected with the AR pathway through multiple mechanisms, and this crosstalk can limit the efficacy of PAM pathway inhibitors (Figure 1). For instance, AKT can affect AR transcriptional function through AR phosphorylation [11,12], while AR can activate the PAM pathway through non-genomic interaction with PI3K [13]. In addition, work by Carver et al. has shown that inhibition of the PAM pathway activates AR signaling by relieving the negative feedback on HER kinases, while inhibition of the AR signaling relieves the PHLPP-mediated negative feedback on AKT in PTEN-deficient PC cells [14]. Similarly to therapies combining PAM and estrogen receptor (ER) pathway inhibitors in breast cancer, concomitant therapies with PAM and AR pathway inhibitors are expected to be more effective than single agent therapies in PC. This hypothesis, supported by several nonclinical studies [14,15,16,17,18,19,20], has provided the basis for clinical trials evaluating inhibitors of the PAM pathway (e.g., samotolisib, ipatasertib, capivasertib, everolimus) in combination with androgen-targeted therapy in PC [4].

Despite the significance of the PAM pathway in PC, evaluations of PAM pathway inhibitors in PC clinical trials have not led to approvals. The majority of the PAM pathway inhibitors previously evaluated in PC clinical trials target single components of the pathway (e.g., capivasertib, which targets AKT). Apparently, when only one component of the PAM pathway is inhibited, several adaptive mechanisms involving other PAM components can reduce the efficacy of single-target inhibitors [21,22,23,24,25,26]. Moreover, the effects of single-target inhibitors of the PAM pathway tend to be limited to specific PC subpopulation, with AKT inhibitors being generally more effective in cells with PTEN loss, and PI3Kα inhibitors in cells with wild-type *PTEN* and mutant *PIK3CA* [27].

By concurrently inhibiting more adaptive mechanisms, a drug targeting multiple PAM pathway components can have improved efficacy, and potentially be effective in a broader PC population, compared to inhibitors targeting single PAM pathway components. In previous nonclinical studies, we showed that gedatolisib, a multi-target PAM inhibitor targeting all class I PI3K isoforms, mTORC1, and mTORC2, induced greater inhibition of the PAM pathway and PAM-controlled functions relative to single-target inhibitors in various cancer cell lines, including breast, endometrial, and prostate cancer. As a result, gedatolisib induced greater anti-proliferative and cytotoxic effects than single-target PAM pathway inhibitors and was similarly effective in cancer cells with or without *PTEN* or *PIK3CA* genetic alterations [28,29,30].

Recent non-clinical studies also showed the benefit of combining gedatolisib with hormone therapy (fulvestrant), with or without a CDK4/6 inhibitor (palbociclib), in hormone-driven, estrogen receptor (ER)-positive breast and endometrial cancer models [28,31]. In the breast cancer models, the gedatolisib/fulvestrant/palbociclib triplet was more effective than the single agents in both treatment-naïve and fulvestrant/palbociclib-adapted cell lines, with or without *PIK3CA* mutations [31]. Consistently, the combination of gedatolisib with hormone therapy and a CDK4/6 inhibitor has shown promising efficacy and safety in a Phase 1b clinical trial [32] and is being evaluated in two ongoing Phase 3 clinical trials (VIKTORIA-1/NCT05501886, VIKTORIA-2/NCT06757634) in ER+/HER2− advanced breast cancer.

Building on the results in breast and endometrial cancer models, the present nonclinical study investigated the hypothesis that combining a multi-target PAM pathway inhibitor like gedatolisib with hormone therapy could also be an effective cancer control strategy in PC. By testing the effects of gedatolisib in combination with darolutamide on multiple cell functions, we found that the gedatolisib/darolutamide combination exerted greater anti-proliferative and cytotoxic effects than the single agents in most of the AR+ PC cell models tested, irrespective of their PTEN status. Importantly, the drug combination was effective in both darolutamide-naïve and darolutamide-adapted cell lines, suggesting that co-treatment with gedatolisib and darolutamide could overcome adaptive resistance to androgen-targeted therapy. These results provide a strong mechanistic rationale for clinical studies evaluating gedatolisib in combination with AR inhibitors in CRPC. A phase 1/2 clinical trial (CELC-G-201) is currently underway to evaluate gedatolisib plus darolutamide in patients with mCRPC previously treated with an AR inhibitor.

## 2. Results

### 2.1. Effects of Gedatolisib in Combination with Darolutamide on Cell Growth

**Figure 2:**
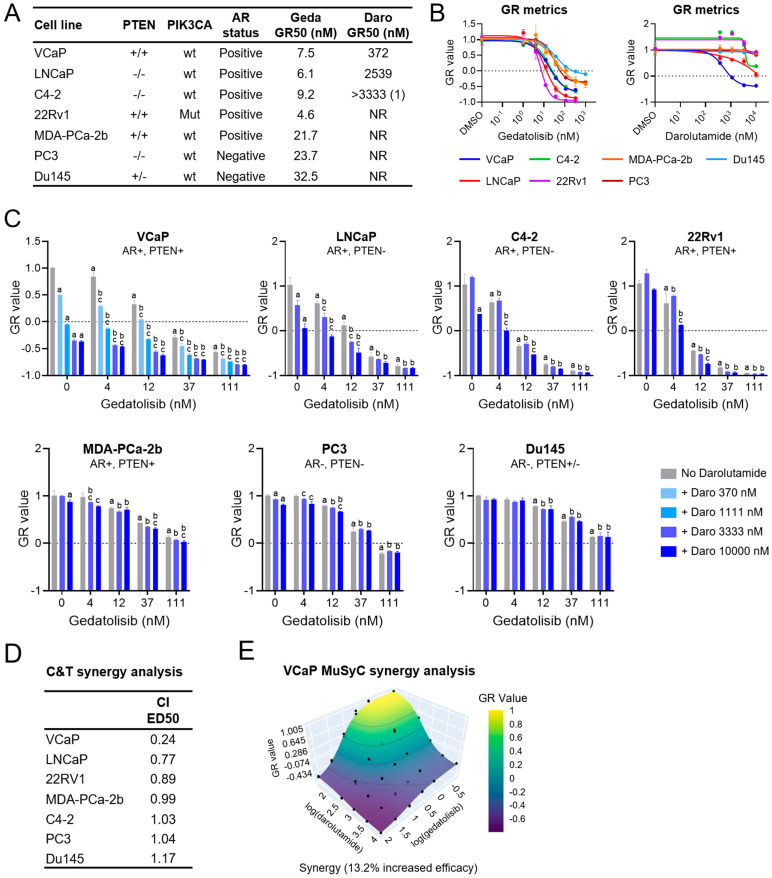
Effects of gedatolisib in combination with darolutamide on cell growth. (**A**) Characteristics of the PC cell lines used in this study, including *PTEN*, *PIK3CA*, AR status, and relative gedatolisib and darolutamide potency (assessed by GR50). (1) GR50 could not be accurately calculated. See Table S1 for GR values. (**B**) Growth rate (GR) metrics analysis showing dose–response curves in PC cells treated with gedatolisib or darolutamide for 72 h. Data represent mean +/− SD (*n* = 2 biological replicates). (**C**) GR metrics analysis of PC cell lines treated with gedatolisib and/or darolutamide at the indicated concentrations for 72 h. Data represent mean +/− SD (*n* = 2 biological replicates). a = *p* < 0.05 for single drugs vs. DMSO, b = *p* < 0.05 for gedatolisib + darolutamide vs. darolutamide, c = *p* < 0.05 for gedatolisib + darolutamide vs. gedatolisib by 2-way ANOVA Fisher test (significance only shown for inhibition). See Table S2 for GR and *p* values. (**D**) Assessment of gedatolisib/darolutamide synergy by Chou-Talalay (C&T) analysis of cell viability (RTGlo MT assay) at the median-effect dose (ED50) after 72 h treatment. Combination index (CI) < 1 indicates synergy; CI > 1 indicates antagonism; CI = 1 indicates additivity. See Table S3 for matrix values. (**E**) MuSyC analysis of GR values after 72 h treatment confirming gedatolisib/darolutamide synergy in VCaP cells. See Table S4 for matrix values. NR = not reached, wt = wild-type; mut = mutant.

To test the effects of gedatolisib in combination with darolutamide on PC cell growth, we used established PC cell lines with different AR and PTEN status (Figure 2A). These cell lines included VCaP and LNCaP, which are widely used as models of AR+, androgen-responsive PC; 22Rv1 and the LNCaP-derived C4-2 cell line, which are used as models of AR+ CRPC; and PC3 and Du145, which do not express AR and are insensitive to androgen [33,34,35]. When grown in medium supplemented with charcoal-stripped serum (i.e., under androgen deprivation conditions), these cell lines showed variable responses to physiological concentrations of dihydrotestosterone (DHT, 1 nM), which significantly promoted cell growth only in VCaP (AR+, PTEN+) and LNCaP (AR+, PTEN−) (Figure S1). To ensure consistent androgen concentration in the medium and evaluate the effects of anti-androgens, all the subsequent cell growth assays were performed in androgen-depleted growth medium supplemented with 1 nM DHT.

The cell lines were first analyzed for their response to increasing concentrations of gedatolisib and darolutamide by calculating growth rate (GR) inhibition from cell viability assessments before and after drug treatment. As we observed in a previous study [30], gedatolisib inhibited cell growth with a GR50 < 40 nM for all cell lines and exerted cytotoxic effects (GR < 0) in all cell lines at the maximum concentration tested (Figure 2A,B). Darolutamide inhibited cell growth in VCaP (AR+, PTEN+) and LNCaP (AR+, PTEN−) cells with GR50 values of 372 and 2539 nM, respectively, and exerted a cytotoxic effect only in VCaP cells (GR = −0.37 at 10 µM). The C4-2 cells (AR+, PTEN−) were less sensitive to darolutamide with a GR50 > 3333 nM and an incomplete dose response curve up to 10 µM. All the other cell lines were completely insensitive to darolutamide up to 10 µM (Figure 2A,B). While gedatolisib growth-inhibitory effects did not correlate with the AR or PTEN status of the cell lines, darolutamide was only effective in AR+ cell lines, regardless of their PTEN status.

Next, the PC cell lines were tested for their growth response to gedatolisib combined with darolutamide. As shown in Figure 2C, the combination of darolutamide and gedatolisib induced greater growth inhibition compared to the single agents in four AR+ cell lines (VCaP, LNCaP, C4-2, and 22Rv1). Interestingly, 22Rv1, which are resistant to darolutamide as a single agent, showed significantly greater growth-inhibition in response to gedatolisib + darolutamide compared to gedatolisib alone, suggesting partial restoration of the darolutamide response. The combination of gedatolisib and darolutamide showed no or very modest benefits in MDA-PCa-2b (AR+), PC3 (AR−) and Du145 (AR−). Synergy analysis with the Chou and Talalay method indicated that gedatolisib and darolutamide inhibited cell growth synergistically (CI < 1) in VCaP, LNCaP, and 22Rv1 (Figure 2D). Synergy was further confirmed in VCaP cells by using the MuSyC method (Figure 2E). Similar results were observed when gedatolisib was combined with enzalutamide, another AR inhibitor (Figure S2).

These results demonstrated that the combination of gedatolisib and darolutamide was more effective than the single agents at inhibiting the cell growth of most AR+ PC cell lines, regardless of their PTEN status. We next focused on the AR+ PC cells to evaluate the effects of the gedatolisib/darolutamide combination on PAM and AR pathway activities as well as cellular functions controlled by these pathways.

### 2.2. Effects of the Gedatolisib/Darolutamide Combination on PAM Pathway and AR Pathway Activities

The AR and PAM signaling pathways are linked by reciprocal feedback loops, whereby inhibition of the AR pathway can promote PAM activity and vice versa, resulting in reduced single agent efficacy in PTEN-deficient PC cells [14,18]. We tested the effects of the gedatolisib/darolutamide combination on AR and PAM pathway activities in AR+ PC cell lines with or without PTEN loss.

**Figure 3:**
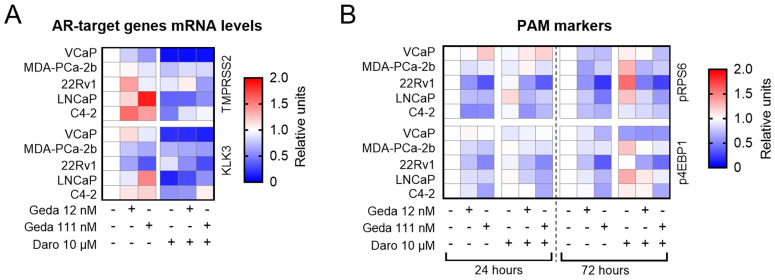
Effects of the gedatolisib/darolutamide combination on PAM pathway and AR pathway activities. (**A**) Quantitative PCR (qPCR) analysis showing relative mRNA levels of two AR-target genes (*TMPRSS2* and *KLK3*) in PC cell lines treated with the indicated drugs for 24 h. (**B**) Flow cytometry analysis showing the relative levels of two markers of PAM pathway activity (p4EBP1 and pRPS6) in PC cell lines treated with the indicated drugs for 24–72 h. In the heatmaps, white indicates no relative change, blue indicates reduction, and red indicates increase in mRNA levels. The values used for the heatmaps are shown in Tables S6 and S7, and represent the mean of 2 biological replicates.

AR pathway activity was assessed by analyzing the transcription of two well-known AR-target genes, *TMPRSS2* and *KLK3* (encoding for the prostate-specific antigen, PSA). As expected, 24 h treatment with 1 nM DHT increased the mRNA levels of both genes in all cell lines analyzed, and co-treatment with darolutamide (10 µM) prevented induction by DHT (see Table S5). In the presence of DHT, gedatolisib (111 nM) increased the mRNA levels of both *TMPRSS2* and *KLK3* in PTEN− cell lines (LNCaP, C4-2, red in heat map), while having no or opposite effects in PTEN+ cell lines (VCaP, MDA-PCa-2b, 22Rv1, white or blue in heat map) (Figure 3A). The increased AR signaling in LNCaP, C4-2 cells treated with gedatolisib is consistent with previous studies reporting that AR signaling is suppressed in PTEN− PC cells and can be reactivated upon inhibition of the PAM pathway [14,18,36]. The combination of gedatolisib and darolutamide reduced the mRNA levels of both genes in all cell lines, regardless of their PTEN status (Figure 3A).

PAM pathway activity was assessed by analyzing the phosphorylation of RPS6 and 4EBP1, two key PAM pathway effectors (Figure 1). Gedatolisib treatment started to decrease pRPS6 and p4EBP1 levels after 24 h, leading to reductions of up to 79% and 65%, respectively, by 72 h in all cell lines (Figure 3B). The addition of darolutamide to gedatolisib did not affect pRPS6 and p4EBP1 levels compared to gedatolisib alone (Figure 3B). The levels of pRPS6 and p4EBP1 were not affected, or very modestly affected, after 24 h treatment with darolutamide, and showed a cell-line dependent increase after 72 h. This increase was counteracted by co-treatment with gedatolisib (Figure 3B).

These data suggest that reciprocal activation of the AR or PAM pathway upon their individual inhibition may be cell line- or context-dependent. Whether or not the treatment with the single agents induced activation of the AR or PAM pathway, the gedatolisib/darolutamide combination effectively inhibited both pathways in all PC cell lines tested, regardless of their PTEN status.

### 2.3. Effects of the Gedatolisib/Darolutamide Combination on Cell Cycle

Our previous studies showed that gedatolisib effectively inhibited cell cycle progression and DNA replication in PC cells [30]. Since AR signaling plays a critical role in controlling cell cycle in PC cells [37], we tested whether the synergistic/additive growth inhibitory effects of the gedatolisib/darolutamide combination were linked to increased inhibition of cell cycle progression and DNA replication.

**Figure 4:**
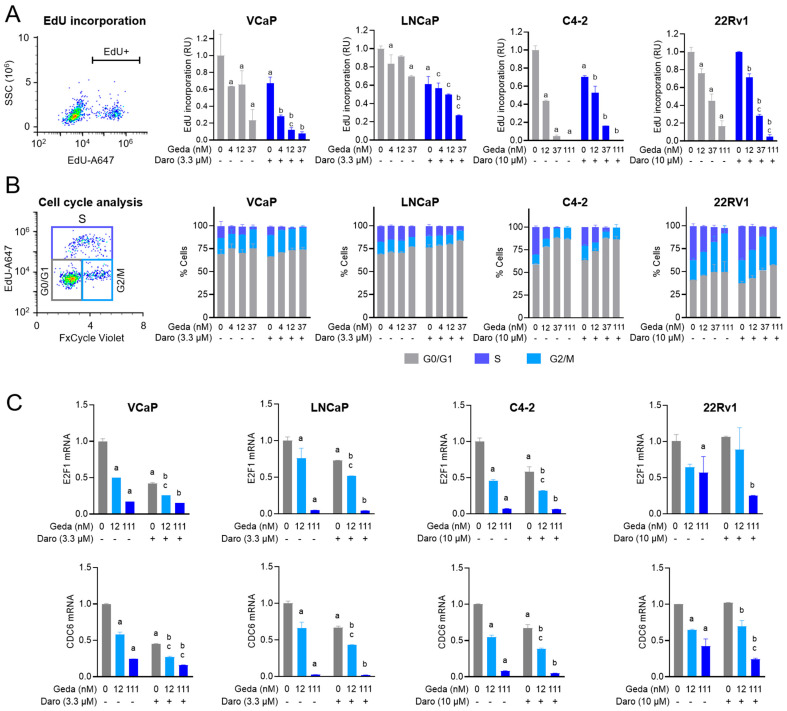
Effects of the gedatolisib/darolutamide combination on cell cycle. (**A**) Flow cytometry analysis of DNA replication by EdU incorporation assay in PC cell lines treated with the indicated drugs for 48 h. An example of flow cytometry plot is shown on the left. Data represent mean +/− SD (*n* = 2 biological replicates). a = *p* < 0.05 for single drugs vs. DMSO, b = *p* < 0.05 for gedatolisib + darolutamide vs. darolutamide, c = *p* < 0.05 for gedatolisib + darolutamide vs. gedatolisib by two-way Anova, Fisher test. (**B**) Flow cytometry analysis of cell cycle phase by EdU incorporation combined with DNA staining with FxCycle Violet. An example of flow cytometry plot is shown on the left. Data represent mean +/− SD (*n* = 2 biological replicates). (**C**) qPCR showing relative *E2F1* and *CDC6* mRNA levels in PC cell lines treated with the indicated drugs for 24 h. Data represent mean +/− SD (*n* = 2 biological replicates). a = *p* < 0.05 for single drugs vs. DMSO, b = *p* < 0.05 for gedatolisib + darolutamide vs. darolutamide, c = *p* < 0.05 for gedatolisib + darolutamide vs. gedatolisib by two-way Anova, Fisher test. RU = relative units. See Tables S8 and S9 for values.

Four AR+ PC cell lines in which the combination of gedatolisib/darolutamide showed synergistic/additive effect by GR metrics analysis were treated with the same drug combination and tested for DNA replication and cell cycle progression by EdU incorporation and FxCycle DNA staining (see flow cytometry plots in Figure 4A,B). The gedatolisib/darolutamide combination inhibited EdU incorporation significantly more than the single agents in VCaP, LNCaP, and 22Rv1 cells, while it did not show any additional benefit in C4-2 cells (Figure 4A). A more in-depth analysis of the cell cycle using both EdU and FxCycle staining further indicated that the gedatolisib/darolutamide combination increased the percentage of cells in G0-G1 and decreased the percentage of cells in S phase, while the drug combination had less pronounced effects on the cells in G2/M (Figure 4B). These results demonstrated that the drug combination mostly arrested cells in G0-G1 by preventing the progression through S phase.

AR has been shown to control cell cycling through direct transcriptional regulation of cell cycle genes such as *E2F1* and *CDC6* [38,39]. Consistent with the GR metrics (Figure 2) and cell cycle (Figure 4A,B) results, treatment with 3.3–10 µM darolutamide significantly reduced *E2F1* and *CDC6* mRNA levels in VCaP, LNCaP and C4-2 cells, while it did not have significant effects in 22Rv1 (Figure 4C). In all cell lines, gedatolisib reduced the mRNA levels of *E2F1* and *CDC6* in a dose-dependent manner. Addition of darolutamide further decreased *E2F1* and *CDC6* mRNA levels (Figure 4C).

These results indicated that the greater growth-inhibitory effects of the gedatolisib/darolutamide combination observed by GR metrics (Figure 2) were associated, in most cases, with a greater inhibition of cell cycle genes and cell cycle progression through the G1-S phase.

### 2.4. Effects of the Gedatolisib/Darolutamide Combination on Cell Death and Apoptosis

**Figure 5:**
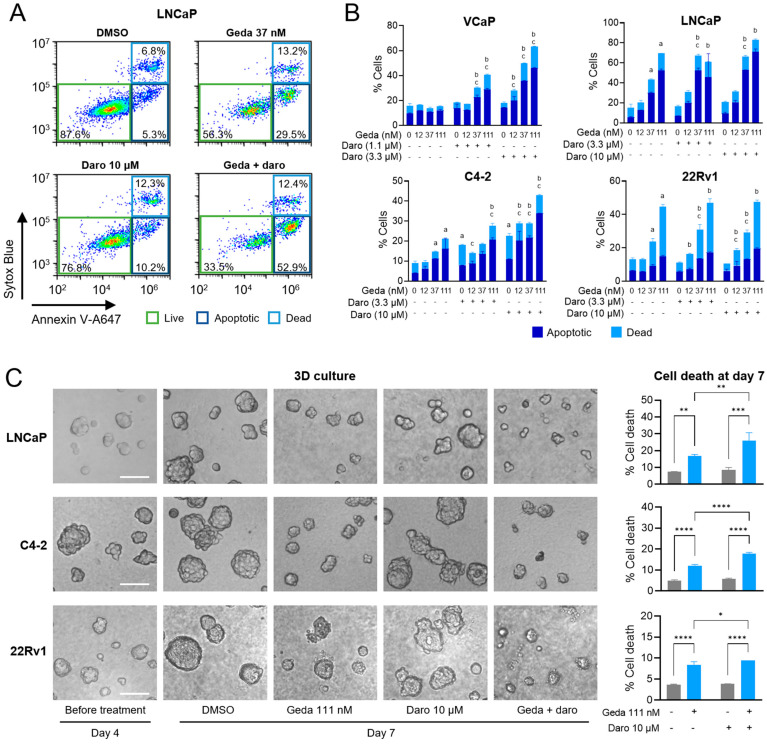
Effects of the gedatolisib/darolutamide combination on cell death and apoptosis. (**A**,**B**) Flow cytometry analysis of cell death and apoptosis by Sytox/Annexin V staining in PC cell lines treated with the indicated drugs for 72 h. The gating strategy to identify dead (Sytox+/Annexin V+) and apoptotic (Sytox-/Annexin V+) cells is shown in (**A**). The quantification of dead and apoptotic cells after the various treatments is shown in (**B**). Data represent mean +/− SD (*n* = 2 biological replicates). a = *p* < 0.05 for single drugs vs. DMSO, b = *p* < 0.05 for gedatolisib + darolutamide vs. darolutamide, c = *p* < 0.05 for gedatolisib + darolutamide vs. gedatolisib by two-way Anova, Fisher test. Statistical significance was calculated based on the sum of dead + apoptotic cells. See Table S10 for values. (**C**) LNCaP, C4-2, and 22RV1 cells were seeded in three-dimensional (3D) culture on basement membrane extract (BME) and allowed to form spheroids for 4 days before a 72 h treatment with DMSO or the indicated drugs (total culture time = 7 days). Representative spheroid images before and after treatment are shown on the left (scale bar = 100 μM). Quantification of spheroids cell death by staining with Sytox Green at the end of the treatment (day 7) is shown on the right. Data represent mean +/− SD (*n* = 2 biological replicates). * = *p* < 0.05, ** *p* < 0.01, *** *p* < 0.001, **** *p* < 0.0001 by two-way Anova, Fisher test. See Table S11 for values.

The combination of PAM pathway inhibitors and darolutamide has been reported to induce apoptosis in androgen-sensitive cells [17]. To test the effects of the gedatolisib/darolutamide combination on apoptotic cell death, we treated VCaP, LNCaP, C4-2, and 22Rv1 cells with each drug, alone or in combination, for 72 h and analyzed the cells by flow cytometry. Overt cell death was detected by staining with Sytox, a DNA stain that can only enter dead cells with a compromised membrane. Apoptosis was assessed in live cells (i.e., Sytox-negative) by staining with Annexin V, which detects phosphatidylserine (PS) exposure on the outer surface of the cell membrane (see flow cytometry plots in Figure 5A). In all cell lines, the gedatolisib/darolutamide combination induced greater cell death and apoptosis relative to the single agents at one or more of the concentrations tested (Figure 5B). The combinatorial effect was especially pronounced in VCaP, LNCaP, and C4-2 cells. Cell death results were also confirmed by staining cells with TMRE, a dye that measures changes in mitochondrial potential and whose decrease is typically observed when cytochrome c is released during apoptosis (Figure S3).

We further tested the effects of the gedatolisib/darolutamide combination in three-dimensional (3D) cultures, which recapitulated features of the extracellular matrix in the tumor tissue. PTEN+ (22Rv1) and PTEN− (LNCaP, C4-2) cell lines, able to grow in 3D culture, were seeded at low density on reconstituted basement membrane and allowed to form spheroids for 4 days before drug treatment for 3 days. At the end of the treatment, the spheroids were imaged and stained with Sytox to assess spheroid cell death. As shown in Figure 5C, gedatolisib reduced spheroid growth and induced cell death in all cell lines as a single agent. Addition of darolutamide to gedatolisib significantly increased cell death, leading to spheroid regression. Consistent with the results in standard 2D culture, the gedatolisib/darolutamide combination increased cell death more effectively in LNCaP and C4-2 cells than in 22Rv1 cells.

These results demonstrated that darolutamide, which has modest effects on cell death as single agent, can increase the cytotoxic effects of gedatolisib in AR+ PC cells, regardless of PTEN mutations. These cytotoxic effects may be especially relevant in cell lines like C4-2 where the gedatolisib/darolutamide combination did not appear to have additive effects on cell cycle.

### 2.5. Effects of the Gedatolisib/Darolutamide Combination on Metabolic Functions

**Figure 6:**
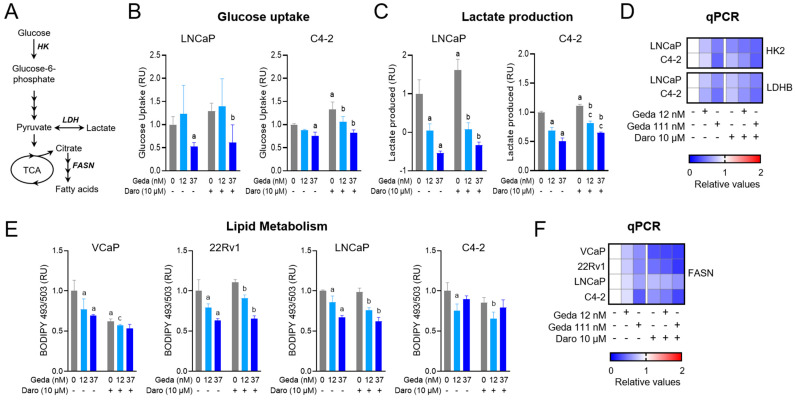
Effects of the gedatolisib/darolutamide combination on metabolic functions. (**A**) Scheme showing selected key steps of glycolysis and fatty acid synthesis. (**B**,**C**) Analysis of glucose uptake and lactate production in PC cell lines treated with the indicated drugs for 4 or 24 h, respectively. The values are relative to DMSO-treated cells (set as 1) and are normalized to cell number (assessed by measuring DNA content using CyQuant assay). Data represent mean +/− SD (see Table S13 for number of replicates). a = *p* < 0.05 for single drugs vs. DMSO, b = *p* < 0.05 for gedatolisib + darolutamide vs. darolutamide, c = *p* < 0.05 for gedatolisib + darolutamide vs. gedatolisib by two-way Anova, Fisher test. (**D**) qPCR analysis showing relative *HK2* and *LDHB* mRNA levels in PC cell lines treated with the indicated drugs for 24 h. (**E**) Flow cytometry analysis of lipid metabolism by BODIPY 493/503 staining in PC cell lines treated with the indicated drugs for 24 h. Data represent mean +/− SD (*n* = 2 biological replicates). a = *p* < 0.05 for single drugs vs. DMSO, b = *p* < 0.05 for gedatolisib + darolutamide vs. darolutamide, c = *p* < 0.05 for gedatolisib + darolutamide vs. gedatolisib by two-way Anova, Fisher test. (**F**) qPCR analysis showing relative *FASN* mRNA levels in PC cell lines treated with the indicated drugs for 24 h. FASN = Fatty acid synthase; HK = Hexokinase; LDH = Lactate dehydrogenase; TCA = tricarboxylic acid cycle; RU = relative units. In the heatmaps, white indicates no relative change, blue indicates reduction, and red indicates increase in mRNA levels. See Tables S13–S15 for values.

PC cells undergo profound metabolic reprogramming during PC progression and function with increased glycolysis and lipid biosynthesis at advanced cancer stages [40,41]. Since both the PI3K pathway and the AR pathway play critical roles in these metabolic adaptations, we tested whether the gedatolisib/darolutamide combination could impact key steps of glycolysis and lipid synthesis (schematically shown in Figure 6A) known to be controlled by these two pathways [9,42].

Glycolytic activity was assessed in LNCaP and C4-2 cell lines by measuring glucose uptake and subsequent conversion of glucose to lactate, an end product of glycolysis. These two cell lines were chosen because they showed higher baseline glycolysis compared to the other AR+ cell lines tested (VCaP and 22Rv1) and therefore represented better models to assess potential inhibition of glycolysis by drug treatments (Table S12). Treatment with gedatolisib significantly reduced in a dose-dependent manner both glucose uptake and lactate production in both cell lines, and the combination with darolutamide did not significantly increase the inhibitory effects of gedatolisib (Figure 6B,C). Consistent with these observations gedatolisib, alone or in combination with darolutamide, decreased the mRNA levels of hexokinase 2 (*HK2*), the enzyme that mediates the first step of glycolysis, and lactate dehydrogenase B (*LDHB*), which is critical for the conversion of pyruvate to lactate (Figure 6D).

Lipids are an important metabolic resource in PC. Substrates for lipid synthesis could arise from citrate and acetate or other by products of glycolysis or glutamine consumption. FASN, an enzyme that catalyzes the final steps of fatty acid de novo biosynthesis, is also a transcriptional target of increased AR activity associated with PC (Figure 6A). Given the likely involvement of PAM and AR in lipid metabolism, we assessed lipid metabolism by measuring changes in BODIPY-stained lipid stores in cells cultured in growth medium with no additional lipid sources. All four cell lines were tested for the effects of gedatolisib and darolutamide on their baseline lipid levels. VCaP, 22Rv1, LNCaP and C4-2 showed similar baseline levels of lipids (Table S12). As shown in Figure 6E, gedatolisib decreased lipid stores compared to baseline levels in all cell lines tested, while darolutamide significantly decreased lipid stores only in VCaP cells. The combination of gedatolisib and darolutamide did not significantly increase the effects of gedatolisib alone on lowering the lipid levels. Without discounting increased lipid utilization as a further cause of lipid stores reduction, we inferred that the decrease in lipid stores upon drug addition could be due to decreased de novo lipid synthesis linked to a reduction in glycolysis. In support of the hypothesis that lipid store reduction is a matter of reduced lipid synthesis from glycolytic intermediates, we observed that gedatolisib, with or without darolutamide, significantly decreased the fatty acid synthase (*FASN*) mRNA levels (Figure 6F).

The data demonstrate that gedatolisib significantly inhibited glycolysis and reduced lipid stores in PC cells lines. Adding darolutamide did not significantly increase gedatolisib’s metabolic effects on glycolysis and lipid stores.

### 2.6. Growth-Inhibitory Effects of the Gedatolisib/Darolutamide Combination in PC Cells Adapted to Darolutamide

Prostate cancer patients often develop resistance to AR inhibitors like darolutamide. To model prostate cancer cells resistant to darolutamide, VCaP (AR+, PTEN+) and LNCaP (AR+, PTEN−) cells were seeded by limiting dilutions and treated with darolutamide (500 nM for VCaP and 5 µM for LNCaP) to isolate single clones adapted to grow in the continuous presence of the drug. A total of seven VCaP-derived clones and five LNCaP-derived clones were expanded and tested for their growth response to AR and PAM inhibitors. Similar results were obtained for the VCaP clones (described hereafter) and the LNCaP clones (shown in Figure S4).

**Figure 7:**
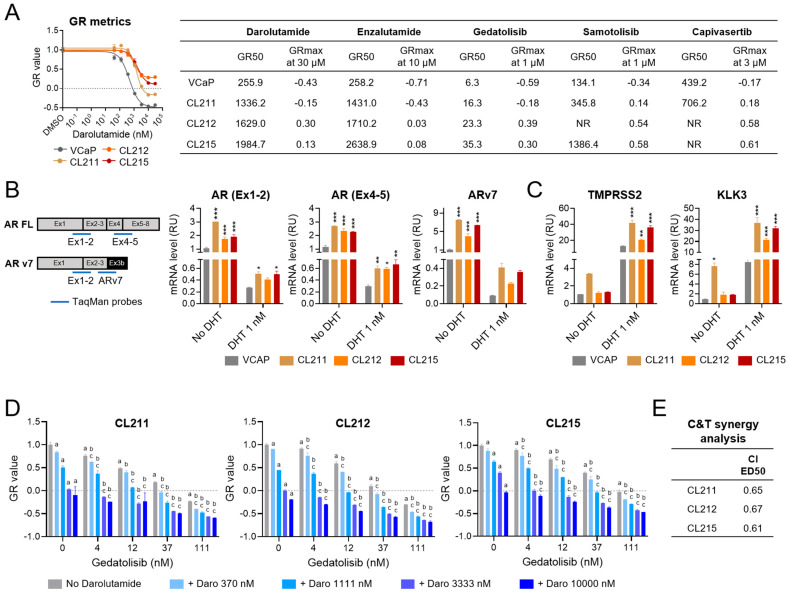
Effects of the gedatolisib/darolutamide combination in PC cells adapted to darolutamide. (**A**) GR metrics analysis showing potency (assessed by GR50) and efficacy (assessed by GRMax) of the indicated drugs in VCaP (parental cell line) and three VCaP-derived clones adapted to grow in the continuous presence of darolutamide (CL211, CL212, CL215). Dose–response curves (DRC) for darolutamide are shown on the left. The DRC data represent mean +/− SD (*n* = 2 biological replicates). (**B**,**C**) qPCR analysis of full-length AR (AR FL), ARv7 splice variant (**B**) and AR-target genes (TMPRSS2 and KLK3) (**C**) in VCaP, CL211, CL212, CL215 cells treated with or without 1 nM DHT for 24 h. The scheme in B shows the Taqman probes used to detect AR FL and ARv7. Data represent mean +/− SD (*n* = 2 biological replicates). * *p* < 0.05; ** *p* < 0.01; *** *p* < 0.001 relative to parental VCaP by two-way Anova, Fisher test. (**D**,**E**) GR metrics analysis (**D**) and Chou-Talalay (C&T) synergy analysis (**E**) in CL211, CL212, CL215 darolutamide-adapted clones treated with the indicated drugs for 72 h. A combination index (CI) < 1 indicates synergy. Data represent mean +/− SD (*n* = 2 biological replicates). a = *p* < 0.05 for single drugs vs. DMSO, b = *p* < 0.05 for gedatolisib + darolutamide vs. darolutamide, c = *p* < 0.05 for gedatolisib + darolutamide vs. gedatolisib by two-way Anova, Fisher test. RU = relative units. See Tables S16–S19 for values.

Analysis of GR metrics showed that the darolutamide GR50 values in the darolutamide-adapted VCaP clones were approximately 3–8 times higher than the GR50 in parental VCaP cells, indicating decreased sensitivity to darolutamide (Figure S5). The three least sensitive clones (CL211, CL212, and CL215, with GR50 = 1336, 1629, and 1985 nM versus GR50 = 256 nM in parental cells, see Figure 7A) were selected for further analysis. These darolutamide-adapted clones were also less sensitive to another AR inhibitor (enzalutamide), as well as to gedatolisib, samotolisib, and capivasertib (Figure 7A and Figure S5).

First, the darolutamide-adapted VCaP clones were tested for the acquisition of potential darolutamide resistance mechanisms. A common resistance mechanism to AR inhibitors is the presence or acquisition of AR alterations, such as AR overexpression or alternative splicing AR variants [43,44]. VCaP cells express both full length *AR* (*AR FL*) and the *ARv7* splice variant, where exons 4–8 are replaced by the alternative exon 3b (Figure 7B) [44]. The absence of the AR ligand binding domain, encoded within exons 4–8, makes the ARv7 variant constitutively active [44]. qPCR with probes recognizing ARv7, AR FL (Ex4-5), or total AR (Ex1-2), showed that the three darolutamide-adapted VCaP clones (CL211, CL212, and CL215) had significantly higher levels of both *ARv7* and *AR FL* mRNAs relative to parental VCaP, both in medium depleted of DHT and in medium supplemented with 1 nM DHT (Figure 7B). Consistently, the VCaP clones also showed increased transcription of two AR-target genes, *TMPRSS2* and *KLK3*, in response to 1 nM DHT, indicating increased AR activity. Interestingly, *KLK3* mRNA (and to a lesser extent *TMPRSS2* mRNA) showed an increase even in the DHT-depleted medium, suggesting DHT-independent AR activity (Figure 7C). Furthermore, additional qPCR experiments showed that the darolutamide-adapted VCaP clones also expressed higher levels of glucocorticoid receptor (*GR*) mRNA (Figure S6), which is another known mechanism of resistance to antiandrogens [45].

Next, the darolutamide-adapted VCaP clones were tested for their response to gedatolisib in combination with darolutamide. GR metrics analysis after a 72 h treatment showed that the gedatolisib/darolutamide combination exerted greater anti-proliferative and cytotoxic effects than the single agents in the three clones tested (Figure 7D). For instance, in clone CL215, 111 nM gedatolisib and 10 µM darolutamide as single agents induced growth inhibitory effects with GR values of −0.02 and −0.03 (mostly cytostatic), and the combination of the two drugs at the same concentrations lowered the GR value to −0.47 (cytotoxic). The increased cytotoxic effects of the gedatolisib/darolutamide combination were also confirmed by flow cytometry analysis of cell death and apoptosis by Sytox/Annexin V staining (Figure S7). Chou and Talalay synergy analysis further showed that gedatolisib and darolutamide had synergistic growth inhibitory effects with CI values < 0.7 in the three clones analyzed (Figure 7E).

These results indicated that the combination of gedatolisib and darolutamide exerted synergistic growth inhibitory effects not only in darolutamide-naïve cells, but also in darolutamide-adapted cells, regardless of their PTEN status.

## 3. Discussion

The connection between the PAM and the AR pathways and the contribution of the PAM pathway to the acquisition of resistance to AR-targeted therapies have provided the rationale for testing combinations of PAM and AR signaling inhibitors in CRPC. Here we show that combining gedatolisib, a multi-target PAM inhibitor, with an AR inhibitor, darolutamide, has synergistic growth-inhibitory effects in AR+ PC cells, regardless of their PTEN status. From a functional standpoint, these effects were associated with cell cycle blockade, induction of apoptotic cell death, and inhibition of metabolic functions. Importantly, the benefits of gedatolisib plus darolutamide were observed both in darolutamide-naïve cells and in cells adapted to grow in the continuous presence of darolutamide, suggesting that this combination could be effective in advanced CRPC tumors with acquired resistance to AR inhibitors.

The reciprocal crosstalk between the AR pathway and the PAM pathway is well established in PTEN-deficient models [14,15,18,19,20]. In vitro and in vivo studies have shown that PTEN loss leads to suppression of AR expression and activity, resulting in depression of PHLPP activity (AKT phosphatase). When the AR signaling is inhibited in PTEN-deficient PC cells, the depression of AR-linked PHLPP levels leads to increased AKT signaling [36]. Furthermore, the inhibition of the PAM signaling activates the AR signaling by relieving the negative feedback to HER kinases [14]. Increased AR signaling in response to gedatolisib could in part explain the combinatorial benefit of adding darolutamide to gedatolisib in PTEN-negative PC cells (e.g., LNCaP). However, gedatolisib did not appear to significantly affect AR transcriptional activity in PTEN+ cells (e.g., VCaP) even if the combination of gedatolisib and darolutamide induced additive/synergistic growth inhibition. Other studies have shown the combinatorial benefits of co-targeting the PAM and AR pathways in PTEN+ PC cell lines [17], but to our knowledge there is no direct evidence of a PAM/AR crosstalk in PTEN+ PC cells. While we cannot exclude that such crosstalk may play a role in PTEN+ cells, the benefit of combining gedatolisib with darolutamide in this cell context is possibly limited to the compounded, yet independent, effects of the two drugs on different critical cell functions.

The combination of gedatolisib and darolutamide affected several cell functions relevant for PC survival and proliferation, regardless of the PTEN status. One of the most significant effects of the gedatolisib/darolutamide combination was the inhibition of cell cycle, which stalled cells in G0/G1 and prevented progression through S phase in both PTEN+ and PTEN− cell lines. The inhibition of cell cycle progression was also paralleled by transcriptional inhibition of key cell cycle genes, such as *E2F1* and *CDC6*, which have been reported to be AR transcriptional targets [38,39]. The AR and the PAM pathways converge on the CDK/Cyclin-RB-E2F axis. The AR pathway can promote cell cycle in androgen-dependent PC cells by affecting multiple G1 regulatory elements that lead to RB phosphorylation and subsequent activation of E2F transcriptional function [37,46]. Moreover, AR can control a distinct transcriptional program and upregulate M-phase cell cycle genes in androgen-independent PC cells [47]. Similarly, the PAM pathway can regulate the cell cycle at various levels, including AKT-mediated control of p21, p27, and cMYC [48], and mTORC1-4EBP1-mediated control of cyclin D1 translation [49]. The concomitant inhibition of PAM and AR pathways by the gedatolisib/darolutamide combination is likely to impact cell cycle at multiple control levels, thus leading to effective cell cycle blockade in androgen-dependent and -independent PC cells, with or without PTEN loss.

In addition to cell cycle blockade, the gedatolisib/darolutamide combination was also effective at inducing cell death in 2D culture and tumor spheroid regression in 3D culture. Cell death was associated with markers of apoptosis, including increased Annexin V and decreased mitochondrial membrane potential, which is often associated with cytochrome c release during apoptosis. Since both the AR and PAM pathway can play a role in mitochondrial function [50,51], the loss of mitochondrial potential could be not only a consequence, but also a cause, of the apoptotic events triggered by the drug combination. The induction of both cytotoxic effects and cytostatic effects (i.e., cell cycle blockade) can explain, at least in part, the overall additive/synergistic growth-inhibitory action of the gedatolisib/darolutamide combination in both PTEN− and PTEN+ cell lines.

The PAM and AR pathways each play a significant role in PC cell metabolism [9,42]. PC cells undergo unique metabolic adaptations to produce enough energy and biomolecules to sustain their increased proliferation. As detected in many other cancers, advanced PC cells are characterized by an increase in glycolysis, which provides additional carbon for anabolic processes and an energy source. Moreover, the citrate produced through excessive glycolysis and the TCA cycle can be utilized for de novo lipid synthesis, which also increases during PC progression [52]. These metabolic adaptations represent vulnerabilities of cancer cells that could be targeted to uncouple biosynthesis demand and the metabolic activities required to meet such demand, which can eventually trigger metabolic catastrophe and cell death [53,54]. Consistent with our previous studies [30], gedatolisib effectively inhibited glycolysis and lipid synthesis in PC cell lines; however, the addition of darolutamide did not increase gedatolisib effects on these metabolic processes. Even if other metabolic activities may be affected by darolutamide, our data suggest that inhibition of the PAM pathway by gedatolisib was the main driver of the metabolic effects induced by the gedatolisib/darolutamide combination. Overall, our functional studies highlight the relative contribution of each drug to the inhibition of different cell functions. Under our experimental conditions, darolutamide mainly affected cell cycling, while gedatolisib appeared to have a much broader effect, stalling cell cycling, reducing cell survival, and disrupting cell metabolism.

We previously demonstrated that gedatolisib inhibited multiple PAM pathway targets and thus exerted greater growth-inhibitory effects than single-target inhibitors in various cancer cell lines [28,29,30]. While gedatolisib is similarly effective in cancer cells with mutated or wild-type *PIK3CA/PTEN*, single-target inhibitors tend to be effective only in subpopulations with specific PAM pathway alterations. For example, Mao et al. describe that PIK3α inhibitors tend to be more effective in *PIK3CA*-mutated, PTEN+ PC cells, while AKT inhibitors tend to be more effective in PTEN-deficient PC cells [27]. The greater and broader efficacy of multi-target PAM inhibitors like gedatolisib may be due to their ability to overcome adaptive mechanisms triggered when only one component of the PAM pathway is inhibited. In addition, gedatolisib inhibits all Class I PI3K isoforms and mTOR with similar, low nanomolar potency (e.g., p110α IC50 at 0.4–0.8 nM regardless of common clinical p110α mutations, mTOR IC50 at 1 nM), while other multi-target inhibitors show uneven potency against their targets (e.g., samotolisib inhibits PI3Kα and mTOR with 6 nM and 165 nM IC50, respectively) [55,56]. Here we present data for the effectiveness of the combination of gedatolisib and AR inhibitors in both PTEN+ and PTEN− PC cells, including PC models adapted to darolutamide. Since more than 40% of PC tumors do not present with PTEN loss [5,7,57], the combination of gedatolisib with AR inhibitors could target a broader PC population than may be indicated for combinations of single-target PAM pathway inhibitors with AR inhibitors. These observations also suggest that PC treatment with gedatolisib and AR inhibitors may not require patient selection based on PTEN status.

Several nonclinical studies reported the benefit of combining AR pathway inhibitors (e.g., bicalutamide, enzalutamide, darolutamide) with single-target (e.g., everolimus, capivasertib, ipatasertib) or multi-target (e.g., BEZ235 and copanlisib) PAM pathway inhibitors [14,15,17,18,19]. These studies, though mostly limited to PTEN-negative cell models, consistently show combinatorial efficacy with respect to cell cycle inhibition and/or induction of apoptosis, two drug effects also induced by the gedatolisib/darolutamide doublet. One study comparing inhibitors of the PAM pathway with different selectivity profiles in combination with darolutamide reported that the combination of the panPI3K inhibitor, copanlisib, with darolutamide exerted some of the most pronounced cytotoxic effects in both PTEN-negative and PTEN-positive PC cell lines [17]. The gedatolisib/darolutamide combination, by targeting mTORC1/2 in addition to PI3K and AR, could be more effective than the copanlisib/darolutamide combination or other combinations targeting fewer PAM pathway components. Side-by-side comparisons of the gedatolisib/darolutamide doublet with other PAM/AR inhibitors doublets would be required to address this question.

A relevant finding of our study is that the gedatolisib/darolutamide combination showed synergistic growth inhibition in AR+/PTEN− (LNCaP) and AR+/PTEN+ (VCaP) darolutamide-adapted cell lines developed to model advanced PC that progressed after treatment with AR inhibitors. Resistance to AR inhibitors is common in advanced PC and could be due to multiple mechanisms, including GR activation, AR overexpression, and constitutively active AR variants [58]. As previously reported for enzalutamide-resistant VCaP and LNCaP cell models [59], we found that adaptation to darolutamide was associated with the increased expression of full-length *AR* in both VCaP and LNCaP darolutamide-adapted clones and *ARv7* in the VCaP clones. Similarly, the darolutamide-adapted VCaP clone also displayed increased *GR* expression. The increased expression of AR, AR variants, and/or GR may provide an explanation for the decreased sensitivity to AR inhibitors in these cell models. Since the darolutamide-adapted clones also became less sensitive to gedatolisib and other PAM inhibitors, other resistance mechanisms may also be involved. For instance, inhibition of the AR signaling in PC cells has been reported to promote epithelial–mesenchymal transition (EMT), which is a known general mechanism of therapeutic resistance [60,61]. However, preliminary experiments showing almost undetectable levels of EMT marker transcripts (ZEB1, SNAI1) in the darolutamide-adapted clones (Table S28) do not seem to support the acquisition of a mesenchymal phenotype.

Hormone-dependent cancers such as prostate, breast, ovarian, and endometrial cancer share the common feature of being driven by sex steroid hormones like androgen and estrogen. This commonality has made these cancers amenable to hormone therapy, which is the standard of care for both hormone-driven prostate and breast cancer. Since dysregulation of the PAM pathway can contribute to hormone therapy resistance, the combination of hormone therapy with PAM pathway inhibitors represents a promising therapeutic strategy in these cancers [3,4,62,63]. The combination of gedatolisib with hormone therapy has shown promising nonclinical efficacy in both breast and endometrial cancer models, including those adapted to long-term hormone therapy [28,31]. The results presented here extend these observations to darolutamide-naïve and darolutamide-adapted PC models, suggesting that a general mechanism may underlie the efficacy of gedatolisib/hormone therapy combinations. In the breast and prostate cancer models, one of the mechanisms appears to be a compounded inhibitory effect on cell cycling, which was driven by both gedatolisib and hormone therapy, alongside metabolic dysregulation and induction of apoptotic cell death, which were predominantly driven by gedatolisib.

The combination of PAM pathway inhibitors with androgen-targeted therapies has been evaluated in several CRPC clinical trials [4]. However, many of these studies reported limited clinical benefit and/or dose-limiting toxicities, and so far, have not led to the FDA approval of any of the combinations tested for CRPC [64,65]. Of note, another pan-PI3K/mTOR inhibitor, samotolisib, has been recently tested in combination with enzalutamide in a Phase 1b/2 mCRPC clinical trial. This combination showed improved PFS versus placebo, but the clinical benefit was less significant in patients with ARv7 and loss of PTEN [66]. The non-clinical data presented here show that the gedatolisib/darolutamide combination is effective in darolutamide-adapted PC cells with increased ARv7, regardless of PTEN status, suggesting that this combination could be effective in this CRPC patient population where others have been less successful.

Preliminary gedatolisib efficacy and safety has been shown in various solid tumors in early phase clinical trials [32,67,68,69,70]. The combination of gedatolisib with hormone therapy and a CDK inhibitor in advanced breast cancer patients with and without *PIK3CA* mutations was shown to be well tolerated and effective [32], and is currently being evaluated in Phase 3 clinical trials (VIKTORIA-1, NCT05501886; VIKTORIA-2, NCT06757634). In addition, gedatolisib in combination with darolutamide is being evaluated in patients with mCRPC previously treated with an AR inhibitor in a Phase 1/2 clinical trial (CELC-G-201, NCT06190899). Initial results from the CELC-G-201 trial have shown favorable preliminary efficacy of the gedatolisib/darolutamide combination and reported no dose-limiting toxicities [71]. The nonclinical results presented here provide a strong rationale for this or other clinical studies assessing gedatolisib in combination with AR inhibitors in CRPC.

## 4. Materials and Methods

### 4.1. Cell Culture

The PC cell lines 22Rv1 (CRL-2505, RRID:CVCL_1045), Du145 (HTB-81, RRID:CVCL_0105), LNCaP (CRL-1740, RRID:CVCL_1379), LNCaP C4-2 (CRL-3314, RRID:CVCL_4782; here referred to as C4-2), MDA-PCa-2b (CRL-2422, RRID:CVCL_4748), PC3 (CRL-1435, RRID:CVCL_0035), and VCaP (CRL-2876, RRID:CVCL_2235) were obtained from ATCC (Manassas, VA, USA), authenticated by STR profiling (ATCC), and tested for mycoplasma. Cells were cultured based on the vendor’s recommendations in medium containing 10–20% FBS (R&D Systems, Minneapolis, MN, USA, Cat# S11550) in a 37 °C, 5% CO_2_ humidified incubator. To ensure consistency between cultures, the same FBS lot was used in this study. The PTEN and AR status of the cell lines was derived from previous studies [35,72]. The *PIK3CA* status was derived from analysis of the Cancer Cell Line Encyclopedia (CCLE, Broad 2019 dataset) through cBioPortal (RRID:SCR_014555, https://www.cbioportal.org/, accessed on 7 July 2022). To develop darolutamide-adapted VCaP and LNCaP clones, VCaP and LNCaP cells were seeded in 96-well plates by limiting dilution to obtain single clones. The cells were grown in growth medium supplemented with darolutamide at concentrations above the GR50 for each cell line (500 nM for VCaP, and 5000 nM for LNCaP) over a period of 3–4 months. The clones that were able to grow in the presence of darolutamide were expanded under the drug selective pressure and archived for further experiments. The cell lines that were selected with darolutamide are referred to as “darolutamide-adapted”, while the cell lines that were not selected with darolutamide are referred to as “darolutamide-naïve” or “treatment-naïve”. The cellular origin of the clones was confirmed by STR analysis. All cell line samples sent to Charles River Laboratories (Wilmington, MA, USA) for testing were confirmed for the absence of contamination by common Mycoplasma species.

### 4.2. Treatments

Gedatolisib (Celcuity, Minneapolis, MN, USA), samotolisib (Cat# S8322), capivasertib (Cat# S8019), darolutamide (Cat# S7559), and enzalutamide (Cat# S1250) (Selleckchem, Houston, TX, USA) were reconstituted in DMSO (Bio-Techne Tocris, Minneapolis, MN, USA, Cat# 3176). For treatments, cells were seeded in androgen-depleted growth medium, i.e., medium supplemented with charcoal-stripped FBS (R&D Systems, Minneapolis, MN, USA, Cat# S11650) instead of standard FBS. The charcoal-stripped and standard FBS lots used in this study had a DHT concentration of 14.1 pg/mL (0.0489 pM) and 133.1 pg/mL (0.461 pM), respectively (analysis by the Clinical Endocrinology Laboratory, UC Davis, CA, USA). For treatments of darolutamide-adapted VCaP and LNCaP clones, cells were seeded in the absence of darolutamide. For flow cytometry and viability experiments, cells were seeded on white 96-well plates; for RNA experiments, cells were seeded in 12-well plates. Plates were coated with ECM proteins as described [30]. The seeding density was optimized for each cell line to prevent over-confluence by the end of the treatment. After overnight attachment, cells were treated with the indicated drugs or corresponding vehicle (DMSO) for the indicated time. Unless indicated otherwise, all treatments were performed in medium supplemented with charcoal-stripped FBS and 1 nM DHT (Sigma-Aldrich, St. Louis, MO, USA, Cat# D-073). The concentrations of gedatolisib and darolutamide tested in this study were chosen to encompass the concentrations reported in plasma of treated patients (gedatolisib > 15–20 nM up to 168 h after treatment with 154 mg; darolutamide between 2.5 and 5 µM up to 8 h after treatment with 600 mg) [70,73].

### 4.3. Growth Rate (GR) Metrics

GR metrics analyses were performed as described in Sen et al. [30] based on the original method by Hafner et al. [74]. Cell viability before and after drug treatments for 72 h was assessed by RT-Glo MT luciferase assay (Promega, Cat# G9712). Luminescence was measured using an Infinite M1000 microplate reader (Tecan, Männedorf, Switzerland). The viability measurements were used to calculate GR values, which were plotted in dose–response curves using PRISM (RRID:SCR_002798, GraphPad Software 10.4.1, Boston, MA, USA). Anti-proliferative effects are indicated by GR values between 0 and 1; cytostatic effects are indicated by GR = 0; cytotoxic effects are indicated by GR values between −1 and 0. Drug potency and efficacy were assessed by calculating the GR_50_ (concentration needed to obtain GR = 0.5) and GR_Max_ (GR value at the maximum concentration tested) from the dose–response curves.

### 4.4. Drug Synergy Analyses

Synergy was assessed using the Chou-Talalay method [75] on CalcuSyn software 2.11 (ComboSyn, Inc., Paramus, NJ, USA). Drug inhibition data from RT-Glo MT luciferase assay was used for calculation of synergy. A combination index (CI) below 1 is considered synergistic, a value at 1 is considered additive, and a value above 1 is considered antagonistic. The combination index is given for ED50 (effective dose). Synergy was also quantified using the Multi-dimensional Synergy of Combinations (MuSyC) method, which distinguishes between different synergy types (e.g., potency, efficacy) [76]. Drug-induced reduction in GR values calculated from GR metric analysis was used as the input for this analysis. This was entered into the MuSyc online application (https://app.duetbiosystems.com, accessed from 11 March 2024 to 24 May 2024). Synergy in terms of efficacy is given as a percent increase in efficacy of the combination of drugs over the drugs used singly.

### 4.5. Analysis of PAM Pathway Activity

PAM pathway activity was assessed by flow cytometry of pRPS6 and p4EBP1, two key effectors of the PAM pathway, as previously described [30]. Cells treated with the indicated drugs for 24–72 h were harvested from 96-well plates by trypsinization. After a wash with PBS, cells were stained with Zombie NIR (Biolegend, San Diego, CA, USA, Cat# 423106), fixed with 1.6% paraformaldehyde (Electron Microscopy Sciences, Hatfield, PA, USA, Cat# 15710), and permeabilized with cold methanol (Sigma-Aldrich, Cat# 179337). After washing, cells were stained with anti-pRPS6-BV421(S235/S236) (Biolegend, clone A17020B, Cat# 608610, 1:50 dilution) and anti-p4EBP1-AF488 (T36/T45) (BD Biosciences, New York, NY, USA, clone M31-16, Cat# 560287, 1:25 dilution) antibodies and run on a Novocyte 3005 (Agilent, Santa Clara, CA, USA) flow cytometer. Data analysis was performed with NovoExpress 1.5.6 (Agilent). pRPS6 and p4EBP1 median fluorescent intensity in live singlets (selected by forward/side scatter and Zombie staining) was used to assess pRPS6 and p4EBP1 levels. An example of the gating strategy is provided in Supplementary Figure S8.

### 4.6. Analysis of Cell Cycle and DNA Replication

Cell cycle and DNA replication were analyzed by flow cytometry as previously described [30]. Cells treated with the indicated drugs for 48 h were incubated with 10 µM 5-ethynyl-2′-deoxyuridine (EdU) (Thermo Fisher Scientific, Waltham, MA, USA, Cat# C10419) for the last 2 h of the treatment and harvested, stained with Zombie NIR, fixed, and permeabilized as described above. EdU incorporation was detected by using the Click-iT EdU Alexa Fluor 647 kit (Thermo Fisher Scientific, Cat# C10419) per manufacturer’s instructions. After washing, cells were stained with 1 μg/mL FxCycle Violet (Thermo Fisher Scientific, Cat# F10347) and run on a Novocyte 3005 (Agilent) flow cytometer. Data analysis was performed with NovoExpress 1.5.6 (Agilent). Live singlets, selected by forward/side scatter and Zombie staining, were gated based on EdU incorporation and FxCycle to assess DNA replication and to identify G0/G1, S, and G2/M phases. If present, cells in sub-G1 and super-G2 were not included in the analysis. An example of the gating strategy is provided in Supplementary Figure S8.

### 4.7. Analysis of Cell Death and Apoptosis

Cells treated with the indicated drugs for 72 h were harvested from 96-well plates by trypsinization. Both attached and floating cells (potentially containing dead/apoptotic cells) were collected for flow cytometry analysis. Dead cells were identified by staining with Sytox Blue, a DNA stain that only enters dead cells with compromised cell membranes. Apoptotic cells were identified by Annexin V staining, which detects phosphatidylserine (PS) exposure on the outer surface of the cell membrane. Additionally, cells were stained with tetramethylrhodamine ethyl ester (TMRE), which measures changes in mitochondrial potential and whose decrease is typically observed when cytochrome c is released from mitochondria during apoptosis [77]. After washing with PBS + 0.5% BSA (Sigma-Aldrich, Cat# A9647) + 0.02% Sodium Azide (G Biosciences, St. Louis, MO, USA, Cat# 786-299) (FACS staining buffer, FSB), cells were incubated with 200 nM TMRE (Abcam, Waltham, MA, USA, Cat# ab113852) for 30 min at 37 °C, washed with FSB, stained with Annexin V-A647 (Biolegend, Cat# 640943) diluted 1:20 in Annexin V binding buffer (Biolegend, Cat# 422201) for 15 min at 4 °C, and finally incubated for 5 min with 5 μM Sytox blue (Thermo Fisher Scientific, Cat# S34857) diluted in Annexin V buffer. Cells were immediately run on a Novocyte 3005 (Agilent) flow cytometer. Data were analyzed with NovoExpress 1.5.6 (Agilent). Cells were first gated by forward/side scatter to exclude cell debris and aggregates, then gated by Sytox Blue, TMRE, or Annexin V/Sytox Blue staining to identify dead cells (Sytox+/Annexin+) and live apoptotic cells (Sytox-/Annexin+ cells or Sytox-/TMRE− cells). Examples of the gating strategies are provided in Supplementary Figure S8.

### 4.8. Metabolic Studies

Glucose uptake was measured using Glucose Uptake-Glo kit (Promega, Madison, WI, USA, Cat# G9711). Cells were seeded in 96-well plates and allowed to incubate for 16 h under culture conditions, and then treated with the indicated drugs for 4 h. After removing the medium, cells were washed with PBS and incubated with 1 mM 2-deoxyglucose (2DG) for 10 min at room temperature. Stop buffer, neutralization buffer and detection reagent were then sequentially added as per manufacturer’s protocol. The plates were incubated for 1 h at room temperature and luminescence read using an Infinite M1000 microplate reader. Cells incubated with PBS, no 2DG, were used for background subtraction. A negative control reaction was performed with culture medium without cells. Glucose uptake measurements were normalized to cell number, which was assessed by measuring DNA content in the same plate using CyQuant assay (Thermo Fisher Scientific, Cat# C7026).

Glucose and lactate were measured in the conditioned media using the Biosen R-line instrument (EKF diagnostics, Cardiff, UK). Cells were seeded in 96-well plates and allowed to incubate for 16 h under culture conditions, followed by drug treatment for 24 h. Glucose consumed and lactate produced were calculated based on glucose and lactate concentrations measured in media with no cells, which was used as baseline control. EKF Diagnostics RUO Multi Standard (Cat#6130-3221), High Standard (Cat# 6130-6025), and Low Standard (Cat# 6130-6015) solutions were used for instrument calibration. EKF Diagnostics Measurements were normalized to cell number, which was assessed by measuring DNA content in the same plate using CyQuant assay (Thermo Fisher Scientific, Cat# C7026).

BODIPY 493/503 (Cayman Chemical Company, Ann Arbor, MI, Cat#25892) was used to measure neutral lipid storage in the PC cells. Cells were seeded in 96-well plates in charcoal-stripped media supplemented with 1 nM DHT. Cells were treated with the indicated drugs for 24 h. No additional source of lipid was provided in the cell media. After drug treatment, neutral lipids were stained by incubating cells with 5 μM BODIPY 493/503 for 30 min at 37 °C. Cells were then harvested from 96-well plates by trypsinization for flow cytometry analysis. Staining with Sytox Blue 5 μM Sytox blue (Thermo Fisher Scientific, Cat# S34857) was used to identify dead cells. Unstained cells were used as negative control. Samples were run on a Novocyte 3005 (Agilent) flow cytometer and analyzed with NovoExpress 1.5.6 (Agilent). BODIPY 493/503 median fluorescence intensity was assessed in Sytox-negative live cells.

### 4.9. Quantitative PCR (qPCR)

Cells for qPCR were seeded in 12-well plates and allowed to incubate for 16 h under culture conditions before 24 h drug and DHT treatment. RNA was extracted using QuickRNA Microprep kit (Zymo, Irvine, CA, USA, Cat# R1051) following manufacturer’s protocol. Nanodrop was used to measure RNA concentrations. cDNA was synthesized using iScript cDNA Synthesis Kit (Bio-Rad, Hercules, CA, USA, Cat# 1708891) and amplified using Taqman probes (Thermo Fisher Scientific) and Applied Biosystems TaqMan Fast Advanced Master Mix (Thermo Fisher Scientific, Cat# 444557) on a QuantStudio3 thermocycler (Thermo Fisher Scientific). The Taqman probes used were *AR* exon 4–5, Hs00171172_m1; *AR* exon 1–2, Hs00907242_m1; *TMPRSS2*, Hs01122322_m1; *KLK3*, Hs02576345_m1; *NDFIP1*, Hs00228968_m1; *FASN*, Hs01005622_m1; *LDHB*, Hs00929956_m1; *HK2*, Hs00892681_m1; *E2F*, Hs00153451_m1; *CDC6*, Hs00154374_m1. The *ARv7* probe was designed based on sequences provided in Marin-Aguilera et al. [78]. Relative changes in mRNA levels were calculated by the Delta/DeltaCt method using *NDFIP1* as the reference gene.

### 4.10. Statistical Analyses

Statistical significance was calculated by Analysis of Variance (ANOVA) as indicated in the figure legends using PRISM (RRID:SCR_002798, GraphPad Software). *p*-values < 0.05 were considered significant at 95% confidence.

## Data Availability

All data are available in the main text or the Supplementary Materials. The datasets analyzed during the current study are available from the corresponding author upon reasonable request.

## Supplementary Materials

The following supporting information can be downloaded at: https://www.mdpi.com/1422-0067/26/24/11810/s1.
