# Supplementary material for: Synergistic Efficacy of Gedatolisib and Darolutamide in Prostate Cancer to Overcome Resistance to Androgen-Targeted Therapy
Source: Int J Mol Sci. 2025 Dec 6;26(24):11810. doi: 10.3390/ijms262411810 (PMC12958000; doi:10.3390/ijms262411810)
Supplement: Supplementary file 1 [file ijms-26-11810-s001.zip › Khan IJMS- micropics-Rev1.pdf]

Original pictures Figure 5C (LNCaP)

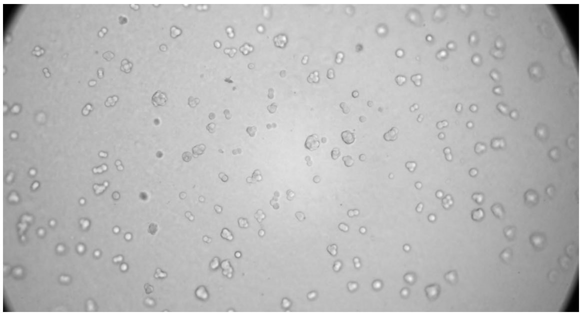

Before treatment

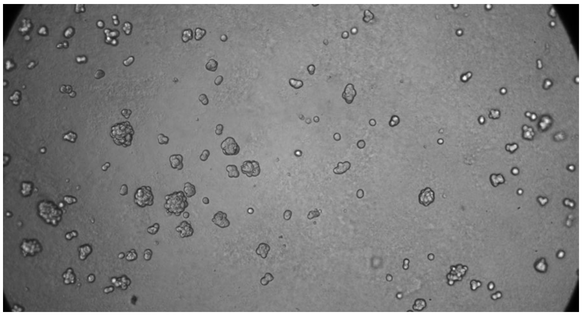

DMSO

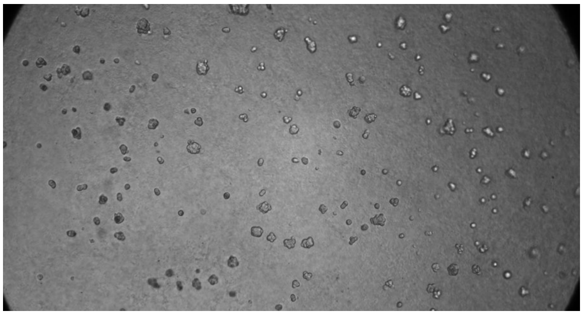

Geda 111 nM

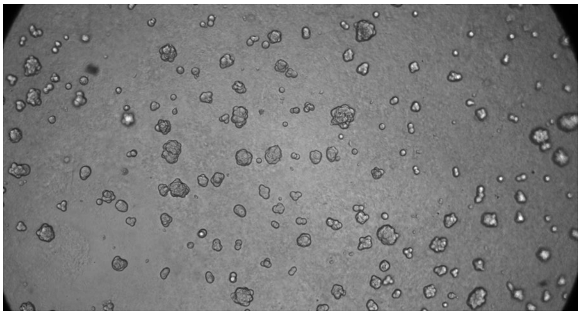

Daro 10 µM

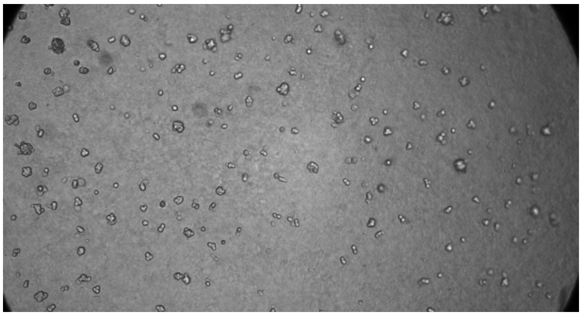

Geda + daro

Original pictures Figure 5C (C4-2)

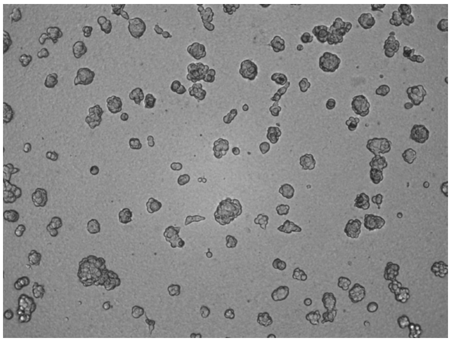

Before treatment

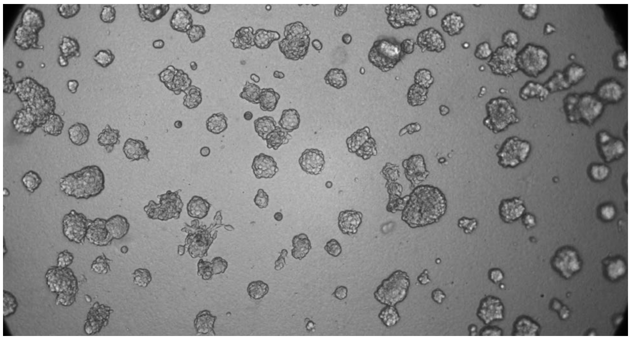

DMSO

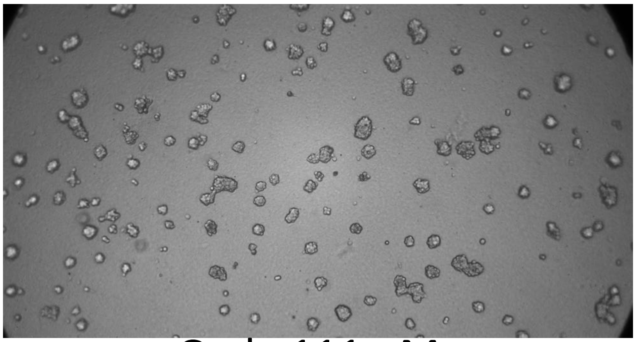

Geda 111 nM

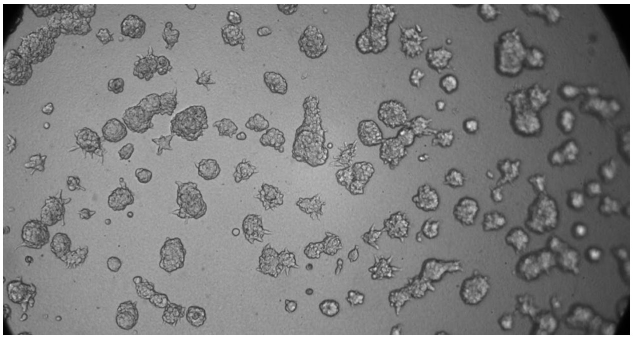

Daro 10 µM

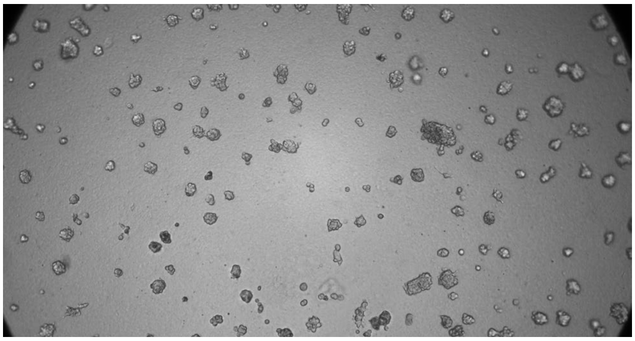

Geda + daro

Original pictures Figure 5C (22Rv1)

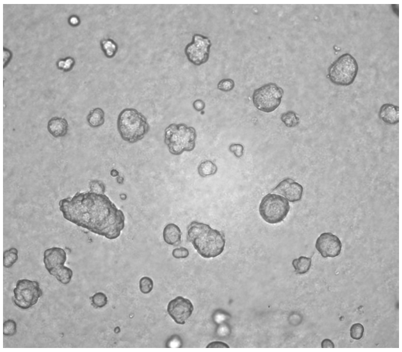

Before treatment

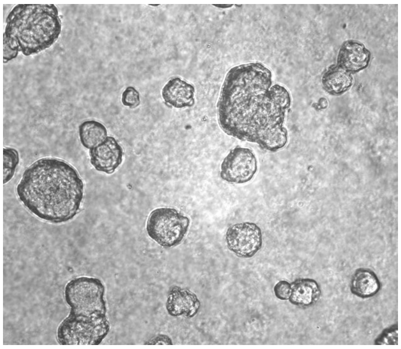

DMSO

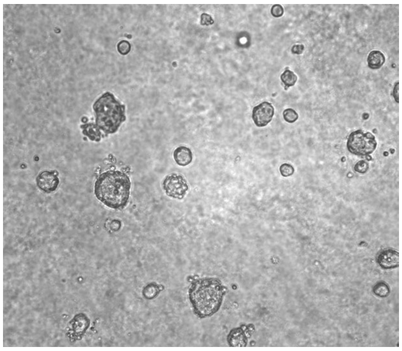

Geda 111 nM

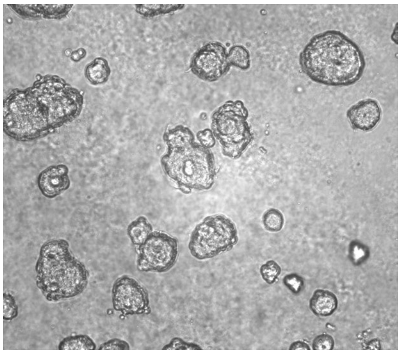

Daro 10 µM

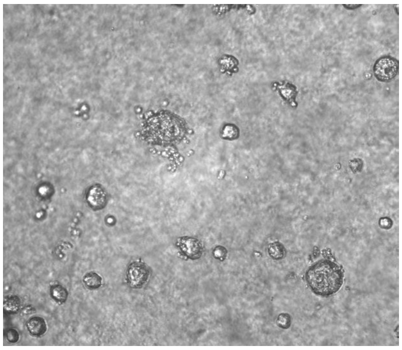

Geda + daro
