# Supplementary material for: Synergistic Efficacy of Gedatolisib and Darolutamide in Prostate Cancer to Overcome Resistance to Androgen-Targeted Therapy
Source: Int J Mol Sci. 2025 Dec 6;26(24):11810. doi: 10.3390/ijms262411810 (PMC12958000; doi:10.3390/ijms262411810)
Supplement: Supplementary file 1 [file ijms-26-11810-s001.zip › Khan_IJMS_Supp Figures_Rev2.pdf]

Supplementary Figures

Synergistic efficacy of gedatolisib and darolutamide in prostate cancer to overcome resistance to androgen-targeted therapy

Salmaan Khan, Jhomary Molden, Charles Iversrud, Donna Mattonen, Stefano Rossetti*, and Lance Laing*

Celcuity, Inc. 16305 36th Ave N, Suite 100, Minneapolis, MN 55446

***Corresponding Author**
llaing@celcuity.com (L.L.)
srossetti@celcuity.com (S.R.)

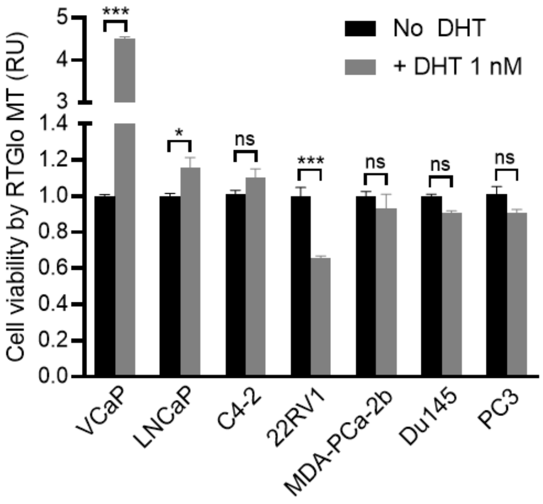

Figure S1. Response to DHT in PC cell lines. Cells were treated with or without 1 nM DHT in growth medium supplemented with charcoal-stripped FBS for 72h. At the end of the treatment, cell viability was assessed by RTGlo MT luciferase assay. Data represent mean +/- SD (*n* = 2) and are relative to no DHT treatment (set as 1). * $p < 0.05$; *** $p < 0.001$; ns = not significant by one-way ANOVA. RU = relative units. See Table S20 for values.

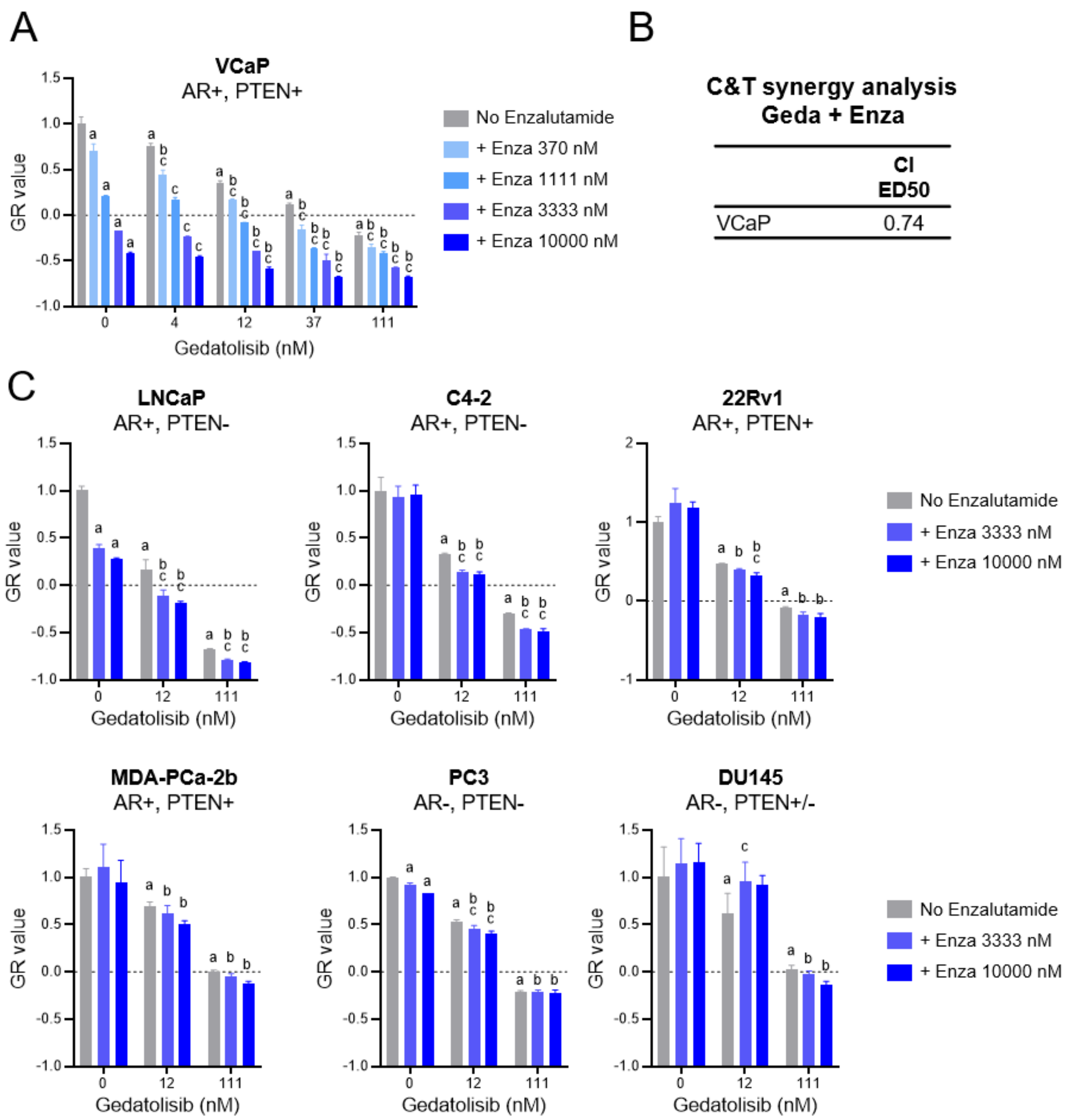

Figure S2. Effects of gedatolisib in combination with enzalutamide on growth rate (GR) of PC cell lines. **A-B.** GR metrics analysis (A) and Chou-Talalay (C&T) synergy analysis (B) in VCaP cells treated with gedatolisib and/or enzalutamide for 72 hours. A combination index (CI) < 1 indicates synergy. **C.** GR metrics analysis in additional PC cell lines treated with gedatolisib and/or enzalutamide for 72 hours. Data represent mean +/- SD ($n = 2$). a = $p < 0.05$ for single drugs vs. DMSO, b = $p < 0.05$ for gedatolisib + enzalutamide vs. enzalutamide, c = $p < 0.05$ for gedatolisib + enzalutamide vs. gedatolisib by 2-way ANOVA Fisher test (significance only shown for inhibition). See Table S21 for values.

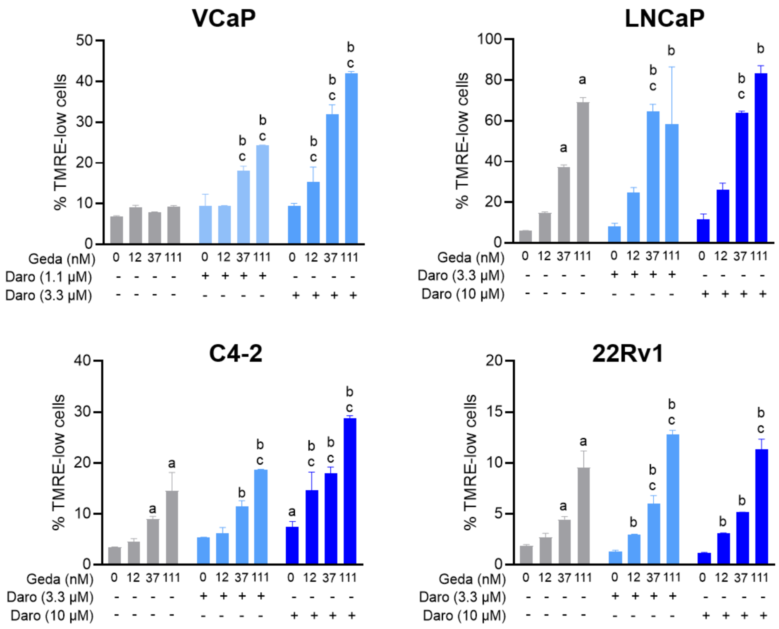

Figure S3. Effects of the gedatolisib/darolutamide combination on apoptosis in PC cell lines. Cells treated with the indicated drugs for 72 hours were stained with Tetramethylrhodamine Ethyl Ester, Perchlorate (TMRE) and analyzed by flow cytometry to assess mitochondrial membrane potential. Loss of mitochondrial potential (TMRE-low cells) is typically associated with apoptosis. Data represent mean +/- SD ($n = 2$); a = p <0.05 for single drugs vs. DMSO, b = $p < 0.05$ for gedatolisib + darolutamide vs. darolutamide, c = $p < 0.05$ for gedatolisib + darolutamide vs. gedatolisib by two-way Anova, Fisher test. See Table S22 for values.

A

	Darolutamide		Gedatolisib	
	GR50 (nM)	GRmax at 30 µM	GR50 (nM)	GRmax at 333 nM
LNCaP	1995	-0.31	6.08	-0.87
CL218	24666	0.46	4.01	-0.47
CL219	2977	-0.28	1.56	-0.89
CL220	17770	0.40	9.75	-0.15
CL221	7151	0.11	5.29	-0.57
CL222	3988	0.14	8.79	-0.45

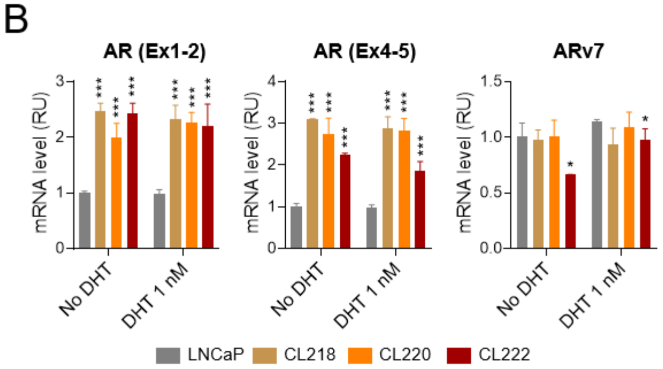

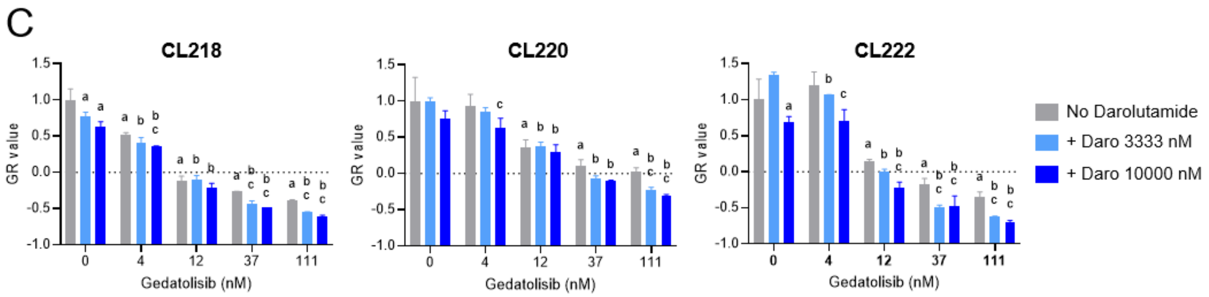

D

C&T synergy analysis

	CI ED50
CL218	0.65
CL220	0.66
CL222	0.75

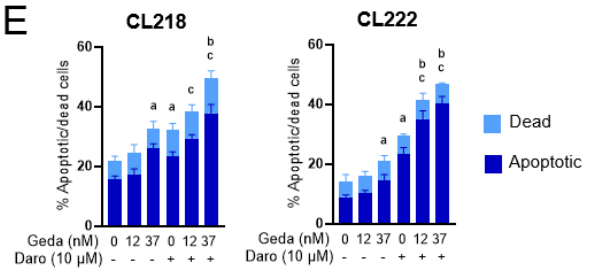

Figure S4. Assessment of drug responses in darolutamide-adapted LNCaP clones. **A.** Comparison of darolutamide, enzalutamide, gedatolisib, samotolisib, and capivasertib potency (assessed by GR50) and efficacy (assessed by GRMax) in parental LNCaP and LNCaP-derived darolutamide-adapted clones. **B.** qPCR analysis of full-length *AR* (*AR FL*), *ARv7* splice variant in LNCaP, CL218, CL220, and CL222 cells treated with or without 1 nM DHT for 24 hours. Data represent mean +/- SD ($n = 2$). * $p < 0.05$; ** $p < 0.01$; *** $p < 0.001$ relative to parental VCaP by two-way Anova, Fisher test. See Table S23 for values. **C-D.** GR metrics analysis (C) and Chou-Talalay (C&T) synergy analysis (D) in CL218, CL220, CL222 darolutamide-adapted clones treated with the indicated drugs for 72 hours. A combination index (CI) < 1 indicates synergy. Data represent mean +/- SD ($n = 2$). a = $p < 0.05$ for single drugs vs. DMSO, b = $p < 0.05$ for gedatolisib + darolutamide vs. darolutamide, c = $p < 0.05$ for gedatolisib + darolutamide vs. gedatolisib by two-way Anova, Fisher test (significance only shown for inhibition). RU = relative units. See Tables S24, S25 for values. **E.** Flow cytometry analysis of cell death and apoptosis by Sytox/Annexin V staining in darolutamide-adapted clones CL218 and CL222 treated with the indicated drugs for 72 hours. The graphs show the percentage of both dead (Sytox+/Annexin V+) and apoptotic (Sytox-/Annexin V+) cells. Data represent mean +/- SD ($n = 3$). a = $p < 0.05$ for single drugs vs. DMSO, b = $p < 0.05$ for gedatolisib + darolutamide vs. darolutamide, c = $p < 0.05$ for gedatolisib + darolutamide vs. gedatolisib by two-way Anova, Fisher test. Statistical significance was calculated based on the sum of dead + apoptotic cells. See Table S26 for values.

A

	Darolutamide		Enzalutamide		Gedatolisib		Samotolisib		Capivasertib	
	GR50	GRmax at 30 μM	GR50	GRmax at 10 μM	GR50	GRmax at 1 μM	GR50	GRmax at 1 μM	GR50	GRmax at 3 μM
VCaP	255.9	-0.43	258.2	-0.71	6.3	-0.59	134.1	-0.34	439.2	-0.17
CL210	1002.3	-0.22	1124.8	-0.52	10.5	-0.12	230.7	0.13	363.1	0.09
CL211	1336.2	-0.15	1431.0	-0.43	16.3	-0.18	345.8	0.14	706.2	0.18
CL217	754.2	-0.35	1154.6	-0.62	11.4	-0.05	285.6	0.24	466.8	0.20
CL212	1629.0	0.30	1710.2	0.03	23.3	0.39	NR	0.54	NR	0.58
CL213	1140.2	-0.07	1272.1	-0.28	18.4	0.14	367.5	0.31	1297.8	0.41
CL214	885.6	-0.05	1067.9	-0.35	13.4	0.13	357.5	0.34	1684.3	0.46
CL215	1984.7	0.13	2638.9	0.08	35.3	0.30	1386.4	0.58	NR	0.61

B

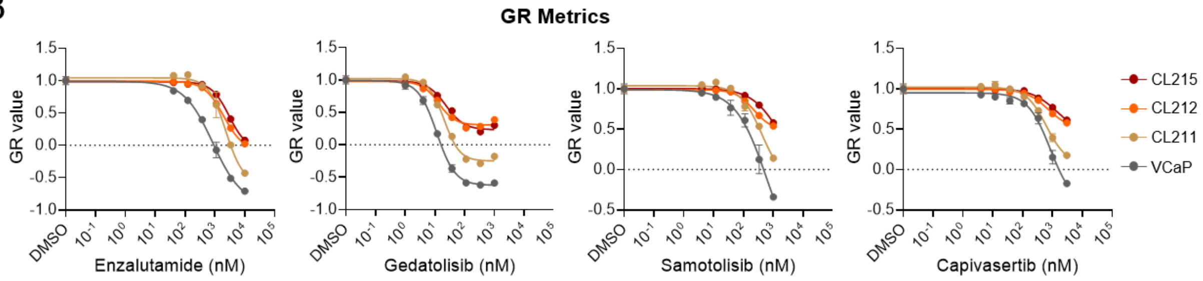

Figure S5. Assessment of drug responses in darolutamide adapted VCaP clones by GR metrics. **A.** Comparison of darolutamide, enzalutamide, gedatolisib, samotolisib, and capivasertib potency (assessed by GR50) and efficacy (assessed by GRMax) in parental VCaP and VCaP-derived darolutamide-adapted clones. **B.** GR dose-response curves for enzalutamide, gedatolisib, samotolisib, and capivasertib in selected darolutamide-adapted clones. See Table S16 for values.

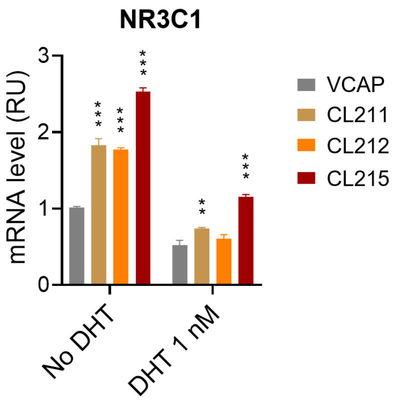

Figure S6. qPCR analysis of *NR3C1* mRNA (encoding glucocorticoid receptor) in darolutamide-adapted VCaP clones treated with or without 1 nM DHT for 24 hours. Data represent mean +/- SD ($n = 2$) and relative to untreated parental cells (set as 1). * $p < 0.05$, **$p < 0.01$, ***$p < 0.001$ relative to parental VCaP by two-way Anova, Fisher est. See Table S17 for values.

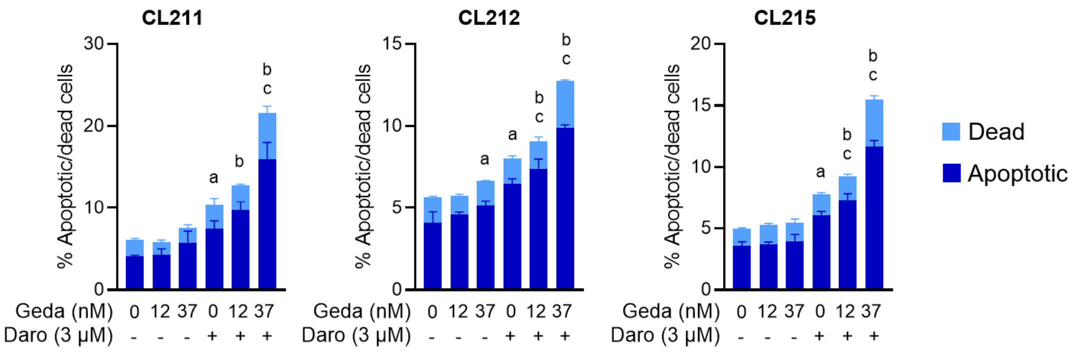

Figure S7. Effects of the gedatolisib/darolutamide combination on cell death and apoptosis in darolutamide-adapted VCaP clones. Flow cytometry analysis of cell death and apoptosis by Sytox/Annexin V staining in darolutamide-adapted VCaP clones (CL211, CL212, CL215) treated with the indicated drugs for 72 hours. The graphs show the percentage of both dead (Sytox+/Annexin V+) and apoptotic (Sytox-/Annexin V+) cells. Data represent mean +/- SD (*n*=2). a = $p < 0.05$ for single drugs vs. DMSO, b = $p < 0.05$ for gedatolisib + darolutamide vs. darolutamide, c = $p < 0.05$ for gedatolisib + darolutamide vs. gedatolisib by two-way Anova, Fisher test. Statistical significance was calculated based on the sum of dead + apoptotic cells. See Tables S27 for values.

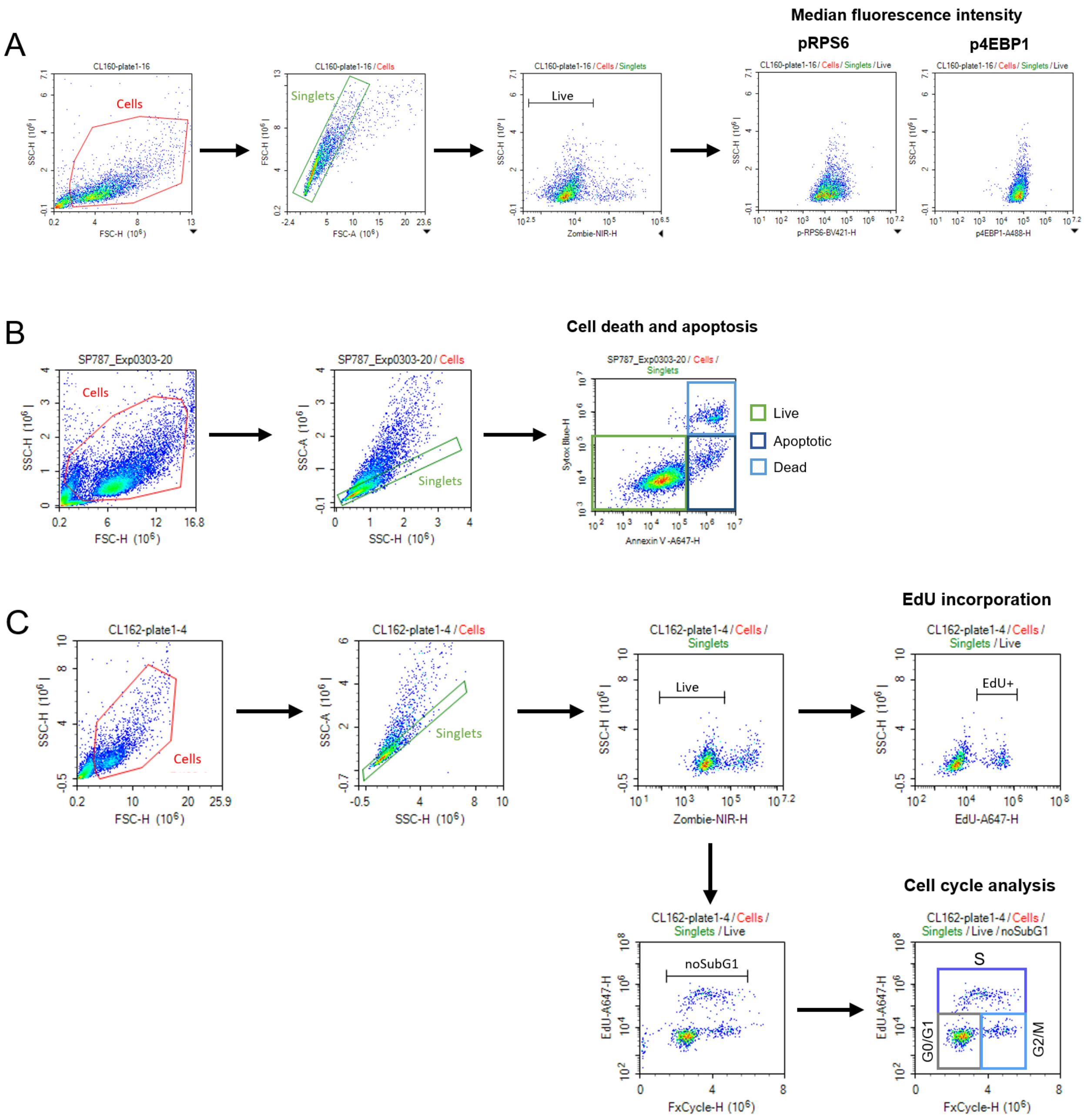

Figure S8. Flow cytometry gating strategies. **A.** pRPS6 and p4EBP1 median fluorescence intensities were assessed by staining with anti-pRP6 and anti-p4EBP1 antibodies in live singlets (Zombie-NIR-low). **B.** Cell death and apoptosis were assessed in all singlets by staining with Sytox Blue and Annexin 5. **C.** For analysis of DNA synthesis, live singlets (Zombie-NIR-low) were gated based on EdU incorporation. For analysis of cell cycle, live singlets were gated based on both EdU incorporation and DNA content (FxCycle violet) to identify cell cycle phases. Cells in subG1 were excluded from the analysis; only the G0/G1, S, G2/M phases were quantified.
